# Dual RNA-seq identifies genes and pathways modulated during *Clostridioides difficile* colonization

**DOI:** 10.1128/msystems.00555-23

**Published:** 2023-08-24

**Authors:** Lucy R. Frost, Richard Stark, Blessing O. Anonye, Thomas O. MacCreath, Ludmila R. P. Ferreira, Meera Unnikrishnan

**Affiliations:** 1 Division of Biomedical Sciences, Warwick Medical School, University of Warwick, Coventry, United Kingdom; 2 Bioinformatics Research Technology Platform, University of Warwick, Coventry, United Kingdom; 3 School of Life Sciences, University of Warwick, Coventry, United Kingdom; 4 RNA Systems Biology Laboratory (RSBL), Department of Morphology, Institute of Biological Sciences, Federal University of Minas Gerais, Belo Horizonte, Minas Gerais, Brazil; Purdue University, West Lafayette, Indiana, USA

**Keywords:** *Clostridioides difficile *infection, *in vitro *gut model, dual RNA-seq, proline-proline endopeptidase

## Abstract

The gastrointestinal pathogen *Clostridioides difficile* is the most common cause of hospital-acquired diarrhea. Bacterial interactions with the gut mucosa are crucial for the establishment of *C. difficile* infection; however, key infection events like bacterial attachment and gut penetration are still poorly defined. To better understand the initial events that occur when this anaerobe interacts with the human gut epithelium, we employed a dual RNA-sequencing approach to study the bacterial and host transcriptomic profiles during *C. difficile* infection in a dual environment *in vitro* human gut model. Temporal changes in gene expression during infection were studied in bacterial and epithelial cells over 3–24 hours. While there were several common differentially expressed bacterial genes across different timepoints after infection, mammalian transcriptional profiles were quite distinct, with little overlap. Interestingly, an induction of colonic receptors for *C. difficile* toxins was observed, along with the downregulation of genes encoding immune response markers. Several cell wall-associated proteins were downregulated in *C. difficile* when associated with host cells, including *slpA*, which encodes the main S-layer protein. Gene function and pathway enrichment analyses revealed a potential modulation of the purine/pyrimidine synthesis pathways both in the mammalian and bacterial cells. We observed that proline-proline endopeptidase, a secreted metalloprotease responsible for cell surface protein cleavage, is downregulated during infection, and a mutant lacking this enzyme demonstrated enhanced adhesion to epithelial cells during infection. This study provides new insight into the host and bacterial pathways based on gene expression modulation during the initial contact of *C. difficile* with gut cells.

**IMPORTANCE:**

The initial interactions between the colonic epithelium and the bacterium are likely critical in the establishment of *Clostridioides difficile* infection, one of the major causes of hospital-acquired diarrhea worldwide. Molecular interactions between *C. difficile* and human gut cells have not been well defined mainly due to the technical challenges of studying cellular host–pathogen interactions with this anaerobe. Here we have examined transcriptional changes occurring in the pathogen and host cells during the initial 24 hours of infection. Our data indicate several changes in metabolic pathways and virulence-associated factors during the initial bacterium-host cell contact and early stages of infection. We describe canonical pathways enriched based on the expression profiles of a dual RNA sequencing in the host and bacterium, and functions of bacterial factors that are modulated during infection. This study thus provides fresh insight into the early *C. difficile* infection process.

## INTRODUCTION

*Clostridioides difficile* is a Gram-positive, anaerobic, spore-forming bacterium and one of the most frequently reported nosocomial intestinal pathogens. Clinical manifestations of *C. difficile* infection (CDI) can range from mild diarrhea to pseudomembranous colitis and toxic megacolon, which are often life-threatening (1). In the United Kingdom, although the number of cases of CDI reported has been declining in recent years, there were 14,249 cases reported between 2021 and 2022 (2). However, in the USA, the number of cases is currently very high, with approximately half a million cases being reported each year, and an estimated 14,000 deaths (3, 4). High recurrence rates of CDI have placed a huge cost burden on healthcare systems worldwide (5).

*C. difficile* produces highly resistant spores which germinate in the presence of bile salts in the gastrointestinal tract (6). Vegetative cells colonize the gut mucosa when the normal gut microbiota is disrupted, usually after antibiotic therapy. Following successful colonization of the gut epithelium, *C. difficile* replicates and secretes toxins, the enterotoxin TcdA, the cytotoxin TcdB, as well as an ADP-ribosylating toxin (CDT) in ribotype 027 strains (7). These toxins are primarily responsible for epithelial barrier disruption, tissue damage, and fluid accumulation during infection (8). In addition to toxins, several other bacterial factors have been identified which have exhibited adhesive properties to host extracellular matrix (ECM) components, epithelial cell layers, or gastrointestinal tissues. These colonization factors include surface layer proteins (SLPs), cell wall proteins (CWPs), flagella, pili, ECM-binding proteins, and secreted enzymes like the proline-proline endopeptidase (PPEP-1) (9 – 14). As seen with other pathogens, *C. difficile* is recognized by Toll-like receptors (TLRs) including TLR-5 and TLR-2 on the host cell surface, initiating innate immune responses against the bacterium (15 – 17). *C. difficile* infection is also accompanied by an increase in the abundance of several proinflammatory cytokines, including interleukins (IL): IL-8, IL-1β, IL-23, IL-33, and tumor necrosis factor alpha (TNF-α) and chemokines, such as C-C motif chemokine ligand 5 (CCL-5) and CCL-2 (18 – 23). Early recruitment of immune cells, such as neutrophils, eosinophils, and interferon gamma (IFN-γ)-producing type 1 innate lymphoid cells, IL-17-producing γδT cells, and IL-33 (19, 24, 25) have also been associated with protection against *C. difficile* infection. Additionally, this bacterium can persist within the gut causing recurrent infections, with sporulation and biofilm formation being key processes contributing to persistence (6, 26).

To understand host pathways activated during infection, host transcriptomic responses have been studied in response to toxins A and B in murine models (27, 28). Studies have also examined the whole-tissue transcriptional responses to vegetative *C. difficile* in a murine infection model, where IL-33 was shown to be upregulated during infection (19). Other studies have examined the bacterial transcriptional responses during infection in porcine and murine models, identifying several known virulence-associated factors and revealing new colonization factors (29, 30). Detailed studies on human intestinal cells have not been possible due to a paucity of *in vitro* models allowing for the coculture of the anaerobic *C. difficile* with oxygen-requiring epithelial cells.

In this study, we investigated the global host and bacterial transcriptomic responses during early *C. difficile* infection, employing a dual environment (anaerobic and aerobic) *in vitro* human gut model (31) and dual RNA sequencing (RNA-seq). We describe here the differential expression of several genes from the host and bacterial cells during the initial 24 hours of *C. difficile* infection. Computational analyses of transcriptomic data identified the canonical pathways enriched at different timepoints after infection, including purine/pyrimidine synthesis, which was enriched in both *C. difficile* and host cells. In *C. difficile*, we observed a downregulation of a proline-proline endopeptidase, a secreted metalloprotease responsible for the cleavage of bacterial cell surface proteins, and further demonstrated that it modulates bacterial adhesion to epithelial cells during infection.

## RESULTS

### Dual RNA-seq of *C. difficile*-infected gut epithelial cells in an *in vitro* gut model

**Fig 1:**
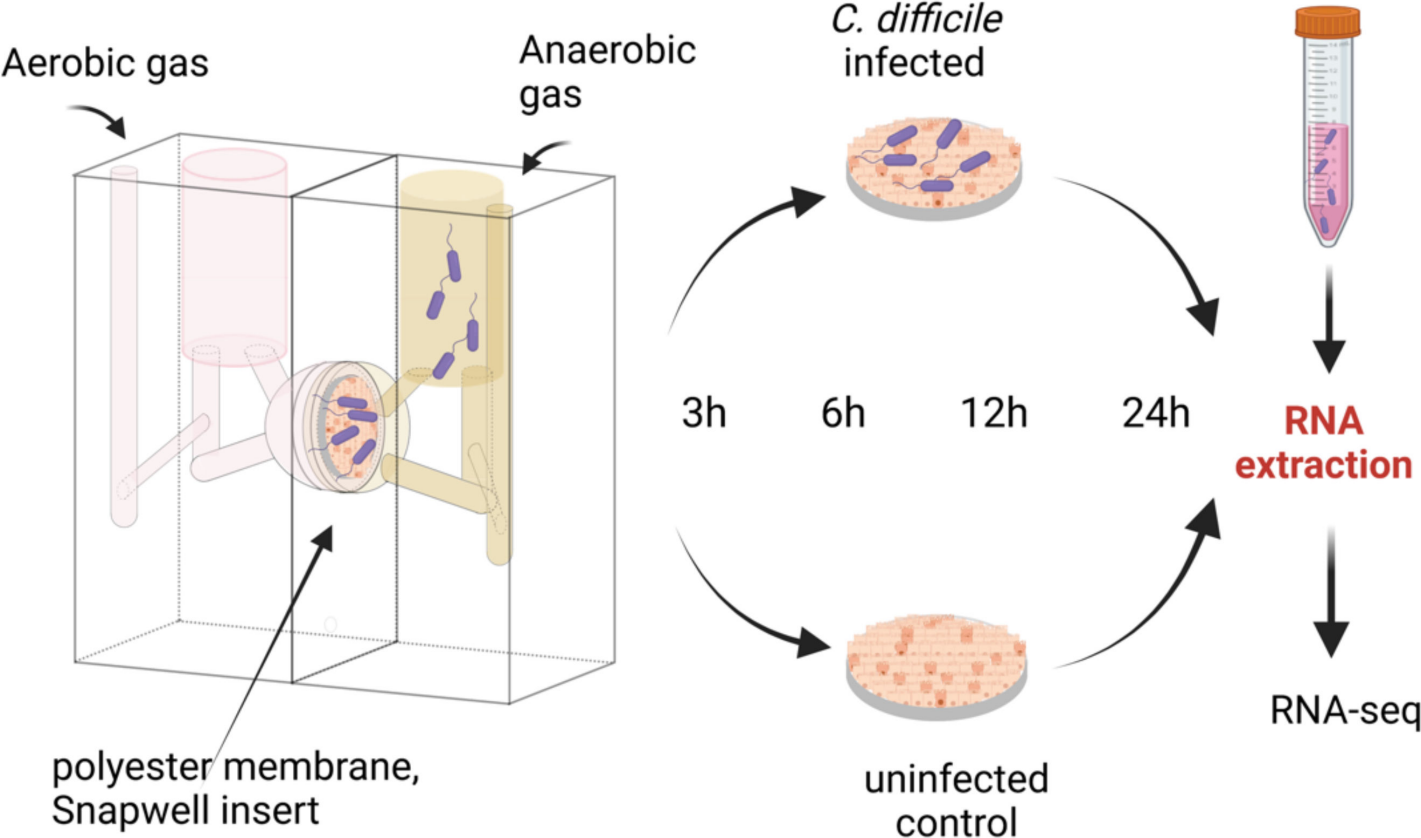
A schematic diagram of the dual RNA-seq experimental setup. An *in vitro* human gut model employing a vertical diffusion chamber (VDC) to facilitate *C. difficile* infection of intestinal epithelial cells. Total RNA was extracted from adhered bacteria and intestinal epithelial cells incubated in the system for 3, 6, 12, or 24 hours, with or without *C. difficile*. A bacterial inoculum grown to the logarithmic phase in pre-reduced DMEM-10 was used as an uninfected bacterial control. Created with Biorender.com.

We have previously optimized an *in vitro* gut infection model where polarized epithelial layers comprising Caco-2 and HT29-MTX cells (9:1) were infected by *C. difficile* R20291 within a vertical diffusion chamber (VDC) system, which enables coculturing in aerobic and anaerobic environments (31). To study transcriptional responses in bacterial and epithelial cells simultaneously, the intestinal epithelial cell layers were infected with *C. difficile* in the VDC at a multiplicity of infection (MOI) of 10 for 3 hours. Extracellular bacteria were washed off, and the cells with adherent bacteria were further incubated until 6, 12, and 24 hours post-infection (Fig. 1). We have reported previously a modest increase is seen in adhered bacteria over 3–24 hours; bacteria produce detectable toxin A by 24 hours in this system, and the transepithelial electrical resistance (TEER) of the host cells also decreases during this timeframe (31). To generate uninfected host control samples, cell layers were incubated in the VDC system for the required times without the addition of *C. difficile* and TEER was measured from cells run in parallel (Table S1). As a control for the bacterial samples, *C. difficile* culture was grown to log-phase in pre-reduced DMEM-10, the same medium used for the infections. Total RNA was extracted from all the infected samples and uninfected controls from 3, 6, 12, and 24 hours of incubation (Fig. 1), and DNA libraries were prepared and sequenced as described in the Materials and Methods section. Sequencing generated 49–64 million reads for the infected samples. While, for all infected samples, over 95% of reads aligned to a concatenated reference containing both the human (GRCh38) and *C. difficile* R20291 genomes, the proportion of reads mapping to the *C. difficile* genome alone was much lower, ranging from 0.06% to 2.06% (Table S2). Although bioanalyzer profiles of purified RNA indicated efficient removal of rRNA, we noted that there was a high proportion of reads mapping to human rRNA on sequencing.

Principal component analysis (PCA) was performed with the human and bacterial RNA-seq data to reduce the dimensionality and visualize variation between samples. All samples with human cells displayed clustering within treatment groups at all timepoints (Fig. S1A). A clear transcriptional shift was observed between uninfected controls and infected samples at all timepoints. We noted changes in the gene expression profiles over the timecourse, not only in infected cell samples but also in the uninfected human cell controls, albeit to a lesser degree. The uninfected 24-hour controls were clustered more closely to the late-stage infected groups than the other uninfected control groups, suggesting a stress response occured in the intestinal epithelial cells incubated in this static system, which is exacerbated over time. For the bacterial samples, PCA plots indicated that biological replicates of 3- and 6-hour infected samples had larger variations in gene expression compared to the other replicates. For this reason, the outliers were completely removed from the analysis. Following their removal, all replicates within samples were clustered together and a clear transcriptional shift from the bacterial culture control was observed for all timepoints (Fig. S1B).

**Fig 2:**
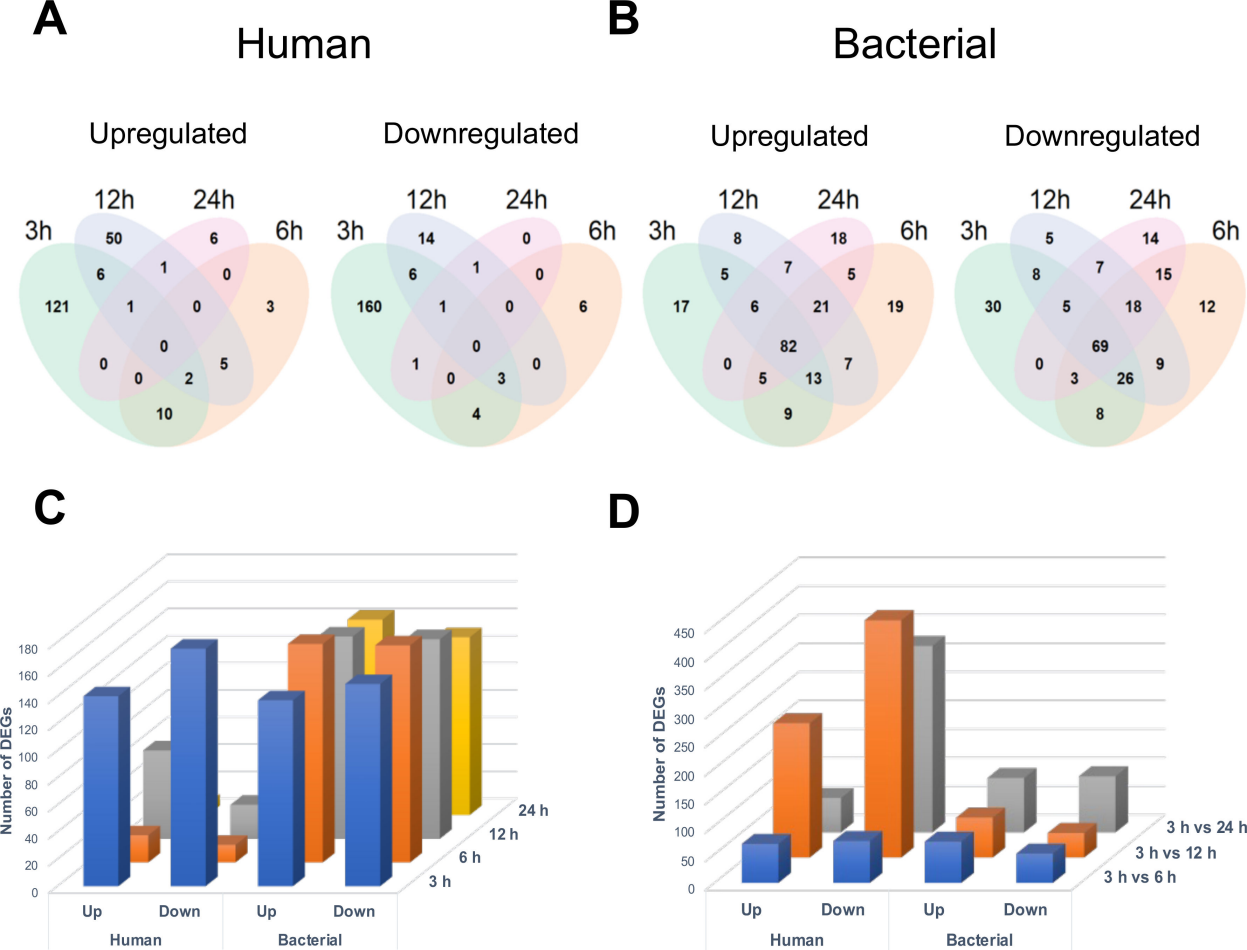
Overall profiles of differentially expressed genes in bacterial and human cells. The DESeq2 (version 1.36.0) R package was used to calculate the differential gene expression profile using a negative binomial distribution model. Differences between groups were considered significantly different when the adjusted *P*-value was below 0.05 and log_2_(FC) was greater than 1 or less than −1 for human and bacterial samples. Venn diagrams of the differentially expressed host (**A**) and bacterial (**B**) genes to visualize genes commonly up- or downregulated between each timepoint when infected samples were compared to the uninfected control samples. (**C**) Numbers of differentially expressed genes (DEGs) at 3, 6, 12, and 24 hours post-infection, as compared to the relevant uninfected controls. (**D**) Number of DEGs as compared between the 3-hour infected samples and the other infected samples at 6, 12, and 24 hours post-infection.

Differentially expressed genes (DEGs) were identified by comparing uninfected controls to infected human samples for each timepoint as described in the Materials and Methods section. A total of 205 and 196 human genes were significantly upregulated and downregulated, respectively [adjusted *P*-value (*P* adj) <0.05 and log_2_(fold change(FC)) greater than 1 or less than −1], with 121 upregulated and 160 downregulated only at 3 hours. No genes were significantly up- or downregulated at all timepoints (Fig. 2A). Thus, the mammalian transcriptional responses induced at each timepoint during infection appear to be distinct, with little overlap in the genes modulated across the different timepoints. The maximum number of human DEGs was also observed when bacteria first interact with cells (3 hours) [*P* adj < 0.05 and a log_2_(FC) >1 or < −1]. 140 genes were upregulated, and 175 genes were downregulated at 3 hours and the changes seen were lower in subsequent timepoints, with an increase seen at 12 hours (Fig. 2C). 70% of the total human DEGs were significant only at 3 hours.

When planktonically grown *C. difficile* was compared with *C. difficile* from cell infections, a total of 222 genes were significantly upregulated, of which 82 were upregulated at all timepoints, and 229 *C*. *difficile* genes were downregulated, of which 69 were significantly downregulated at all timepoints (Fig. 2B; Fig. S2). In this case, there were several bacterial DEGs that were common across 3–24 hours. Among the bacterial DEGs, there were 137 upregulated and 149 downregulated genes identified at 3 hours, but many of these genes stayed altered until 24 hours (Fig. 2C).

**Fig 3:**
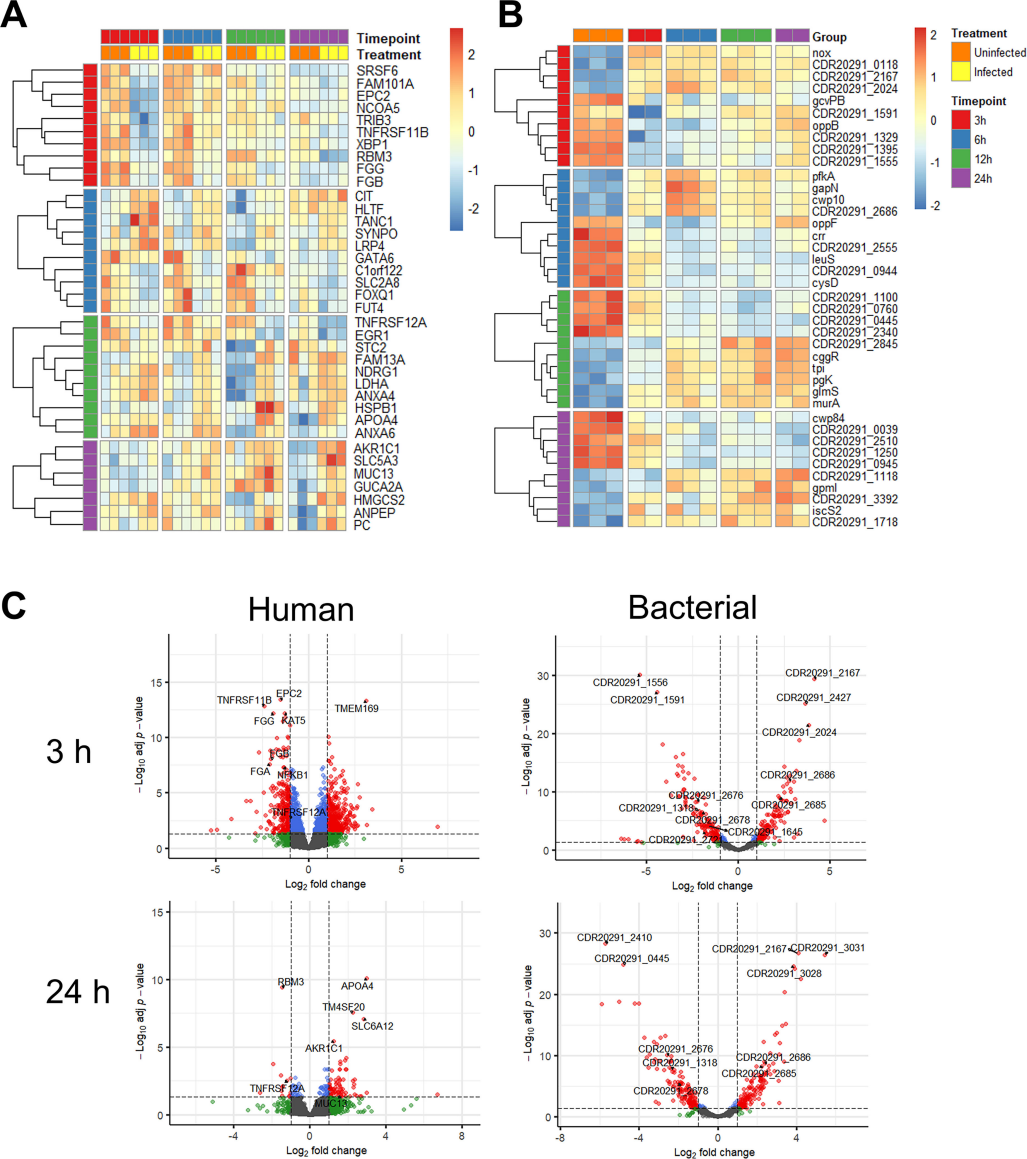
Differentially expressed host and bacterial genes. Heatmaps generated using the “pheatmap” R package showing the top 10 most significant DEGs at each timepoint as compared to the respective uninfected control for human (**A**) and bacterial (**B**) genes. (**C**) Volcano plots to visualize the distribution of human and bacterial genes of uninfected controls versus infected samples at 3 and 24 hours after infection. Significant DEGs can be visualized as having a log_2_(FC) greater than 1 or less than −1 and *P* adj < 0.05 (red dots). Labels and arrows point out selected highly significant DEGs.

The expression levels of the top 10 human and bacterial genes (ranked by *P* adj value), at different times after infection compared to the respective uninfected controls were visualized with heatmaps (Fig. 3A and B), and the complete lists of DEGs compared to uninfected controls are included in Table S3. Volcano plots generated with the ‘EnhancedVolcano’ R package show the distribution of all DEGs at each timepoint for human and bacterial genes in the control vs infection analyses (Fig. 3C; Fig. S3).

A comparison was also made across timepoints to understand the dynamic changes in gene expression. Time-dependent analysis was performed by comparing each timepoint to 3 hours after the removal of genes changing in the uninfected controls (Fig. 2D; Tables S4 and S5); this showed changes occurring over time during infection, with maximal changes (with more genes downregulated) observed between 3 and 24 hours. The top 10 DEGs when compared across different timepoints 3 hours vs 6 hours, 3 hours vs 12 hours, and 3 hours vs 24 hours, after removing genes changing in the uninfected controls, are included in Table 1. The distribution of DEGs by time-dependent analysis is shown in volcano plots in Fig. S4.

**TABLE 1:** Top 10 most significantly different human genes changing at different times compared to 3 hours after infection

Time after infection (hours)	Gene	log_2_(FC)	*P* adj
3 vs 6	BCL6	−1.4263	2.48E-13
	PPRC1	1.461462	7.74E-12
	LCA5	−1.70836	3.39E-09
	CITED2	−1.65225	2.90E-08
	JARID2	−1.00072	7.70E-08
	BEND3	1.47559	1.28E-07
	HGNC:9982	1.153837	1.40E-07
	EPC2	1.230927	1.62E-07
	MYC	1.188266	3.41E-07
	FZD4	−1.48643	5.72E-07
3 vs 12	PHLDB2	−2.31212	8.69E-26
	NOP16	1.871325	4.35E-17
	CDC14A	−2.52359	4.35E-17
	CPLX1	2.625903	3.00E-16
	AMOT	−1.63245	6.42E-15
	PDZD7	−2.25312	9.95E-15
	CITED2	−2.06608	1.69E-14
	FZD4	−2.02591	2.55E-14
	LCA5	−1.89066	8.22E-13
	ZNF608	−1.73279	2.49E-11
3 vs 24	GNRH2	−3.1352	7.45E-21
	ARL4C	−1.8818	7.45E-21
	CCND3	−2.44675	1.67E-18
	REEP2	−2.94226	2.10E-17
	MSR1	−1.98428	5.15E-16
	PDZD7	−2.24464	1.30E-13
	EHD1	−1.38567	1.09E-11
	MYLIP	−1.62791	1.34E-11
	BCAS4	−2.46879	1.51E-11
	VDR	−1.66743	4.92E-11

### Host responses to *C. difficile* attachment and multiplication

**Fig 4:**
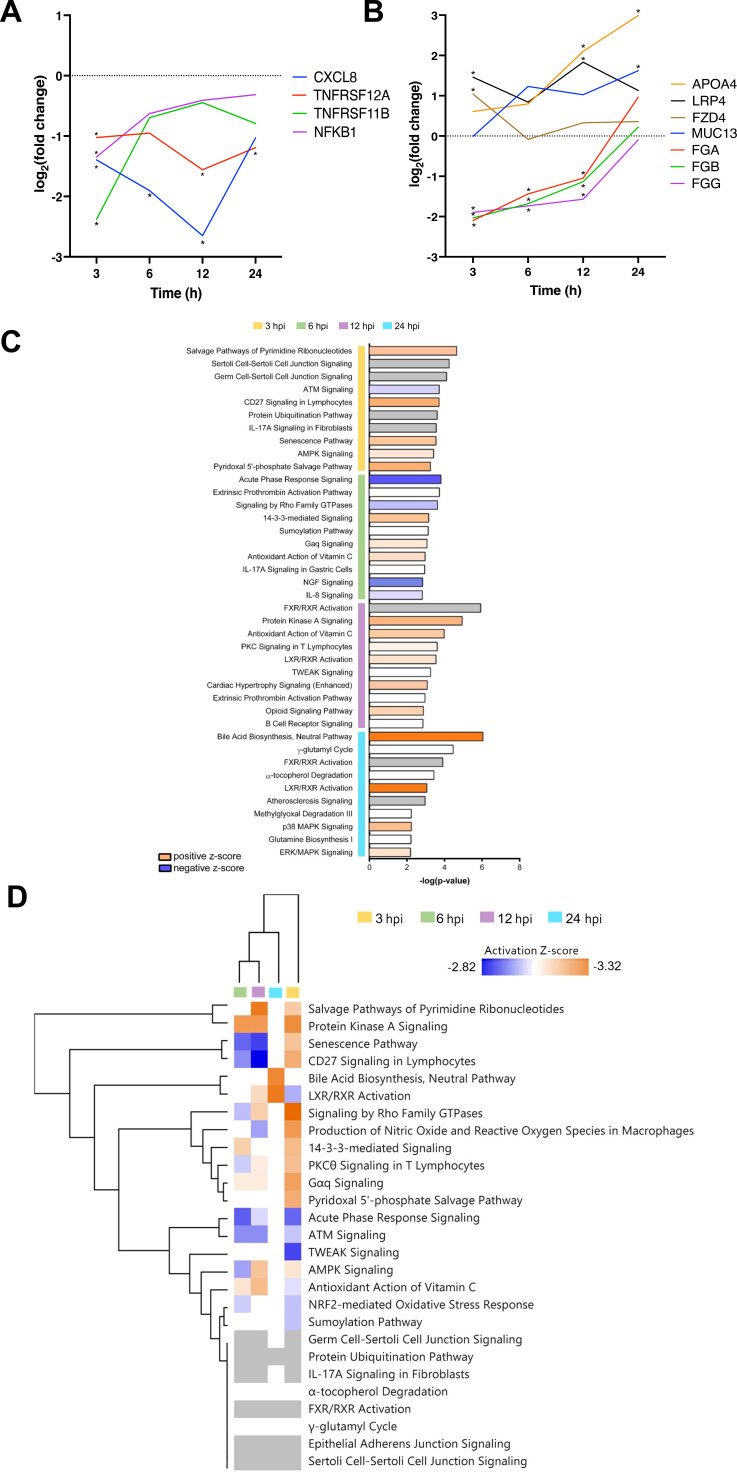
Host pathways and single-gene expression profiles during infection. Host single-gene expression profiles of selected interesting genes (**A**) and immune response genes (**B**) differentially expressed during *in vitro C. difficile* infection. Log_2_(FC) of selected genes were calculated between the expression levels of uninfected controls and infected samples at each timepoint. All selected genes were significantly differentially expressed in at least one timepoint, indicated with an asterisk. (**C**) The Z-scores of the top 10 most significantly enriched pathways at each timepoint. (**D**) Heat map of top significantly enriched pathways. The up- (orange) or downregulation (blue) of each pathway is indicated by the Z-score. Hierarchical clustering was used to indicate the distances in the gene expression profiles between the timepoints.

Among the large number of mammalian genes induced in infected cells compared to uninfected controls, one of the most upregulated genes at 12 and 24 hours post-infection was apolipoprotein A-IV (*APOA4*) [3.08 log_2_(FC) at 24 hours, *P* adj = 3.14E-09], an apolipoprotein primarily produced in the small intestine (Fig. 4A) (32). The gene for mucin 13 (*MUC13*), a transmembrane mucin glycoprotein, which is highly expressed on the surface of mucosal epithelial cells in the small and large intestines (33, 34), was upregulated [log_2_(FC) > 1] at 6, 12, and 24 hours post-infection, but this upregulation was only statistically significant at 24-hours post-infection. Interestingly, the low-density lipoprotein receptor–related protein 4 (*LRP4*), a protein involved in the inhibition of Wnt signaling (35), was significantly upregulated at 3 and 12 hours after *C. difficile* infection (Fig. 4A). The frizzled-4 protein (*FZD4*), a receptor involved in the Wnt/β-catenin canonical signaling pathway, was significantly upregulated at 3 hours after infection [1.56 log_2_(FC), *P* adj = 0.0005], although it did not show any changes in expression at other timepoints (Fig. 4A).

Comparing infected and uninfected cells, one of the most significantly differentially expressed genes at 3 hours after infection was the tumor necrosis factor receptor superfamily member 11B (*TNFRSF11B*). *TNFRSF11B* encodes osteoprotegerin, a receptor which binds to TNF-related apoptosis-inducing ligand (TRAIL) and was downregulated with a log_2_(FC) of −2.45 and *P* adj value of 1.12E-14 (Fig. 4B) (36). Tumor necrosis factor receptor superfamily member 12A (*TNFRSF12A*), known as fibroblast growth factor–inducible immediate-early response protein 14 (Fn14), was significantly downregulated at 3, 12, and 24 hours post-infection [−2.478 log_2_(FC) at 3 hours, *P* adj = 1.12 E-14] (Fig. 4B) (37). Surprisingly, the gene for IL-8 (*CXCL8*), a chemokine, strongly associated with *C. difficile* infection was significantly downregulated at 3, 6, and 12 hours post-infection (31, 38). Also among the highly downregulated genes at 3, 6, and 12 hours post infection were the fibrinogen alpha, beta, and gamma chain genes (*FGA*, *FGB,* and *FGG*) [~1.95 log_2_(FC), *P* adj < 2.66 E-06]. However, no differential expression was observed at 24 hours after infection (Fig. 4B). Thus, several interesting genes that are involved in a range of cellular pathways are regulated during the initial phases of *C. difficile* infection.

Comparing the time-dependent changes between 3 hours and different times after infection (Table 1), several genes like *FZD4*, *LPR4*, and *MUC13* which were upregulated compared to the uninfected controls were also upregulated over time. Additionally, other mucin genes like *MUC12* [1.062 log_2_(FC), *P* adj = 0.024], and other FZD proteins FZD5 [−1.187 log_2_(FC), *P* adj = 3.19 E-07], were upregulated at 12 and 24 hours when compared to 3 hours. Matrix metalloprotease-1 (*MMP1*), which encodes an interstitial collagenase, was upregulated when compared between 3 hours and different times, although when compared to control uninfected samples, this gene was significantly downregulated at each timepoint.

To investigate host cellular processes and pathways which were modulated during *C. difficile* infection, a pathway enrichment analysis was conducted. Overall, the most significantly modulated pathways included the neutral bile acid synthesis pathway (24 hours), the farnesoid X receptor (FXR)/retinoid X receptor (RXR) activation pathway (39), which has a role in the metabolism of bile acids (12 hours) and salvage pathways of pyrimidine ribonucleotides, which are responsible for nucleotides’ recycling from DNA and RNA breakdown (3 hours) (Fig. 4C and D). At 3 hours, the salvage pathways of pyrimidine ribonucleotides had a positive Z-score, suggesting a potential activation during infection, despite some of the genes in the pathway being downregulated. Some other interesting, enriched pathways identified for 3 hours post-infection included Sertoli and germ cell-Sertoli cell junction signaling, CD27 signaling in lymphocytes, IL-17A signaling in fibroblasts, and 5′-AMP-activated protein kinase signaling. At 6 hours after infection, acute phase response signaling, which is involved in the alterations of gene expression and metabolism in response to inflammatory cytokines, had a negative Z-score, indicating that this enriched pathway is potentially inhibited during *C. difficile* infection. Other pathways enriched in the data from 6 hours post infection include the extrinsic prothrombin activation, signaling by Rho GTPases, IL-17A signaling in gastric cells, and IL-8 signaling pathways. At 12 hours post-infection, the most enriched canonical pathways were the protein kinase C signaling in T lymphocytes, TNF-like weak inducer of apoptosis, extrinsic prothrombin activation pathway, and B-cell receptor signaling, and at 24 hours, enriched pathways include FXR/RXR and LXR/RXR activation, p38 MAPK (mitogen-activated protein kinase), and extracellular signal-regulated kinase/MAPK signaling pathway. Comparing the different timepoints, we could identify several pathways that are predicted to be activated in all timepoints post-infection, including the protein kinase A, IL-17, and cell junction signaling pathways (Fig. 4D).

### Bacterial responses to *C. difficile* infection

There were several interesting differentially expressed *C. difficile* genes identified in this study which have roles across many cellular processes, including virulence, colonization, and metabolism. As expected, “housekeeping” genes including *adk*, *rpoC*, and *trxA* did not change significantly in bacterial cells when compared to the control or across time.

**Fig 5:**
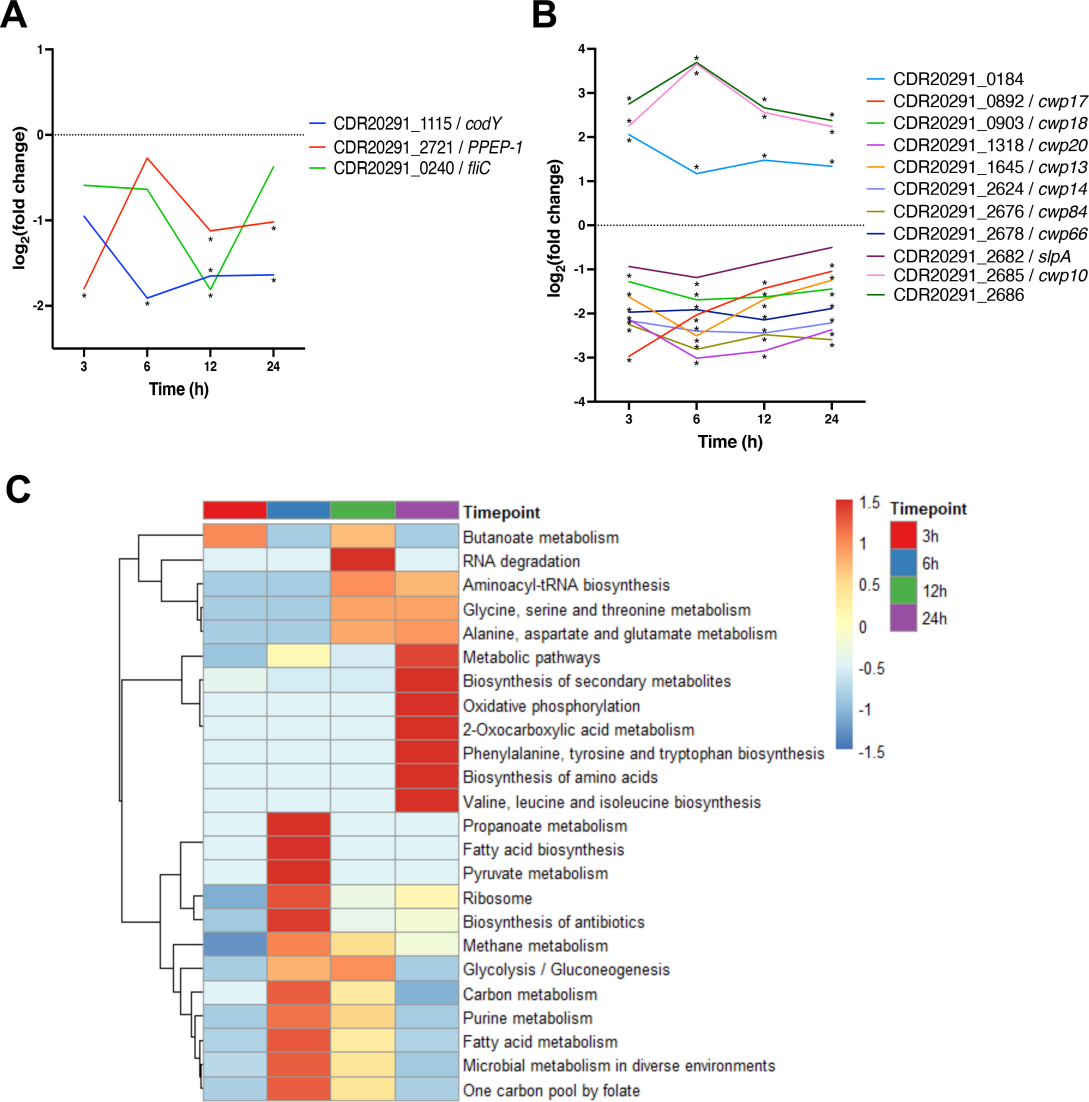
Bacterial genes and pathways change during infection. Single-gene expression profiles of selected *C. difficile* cell-surface proteins (**A**) and virulence-associated genes (**B**) at different timepoints of infection. Statistical significance is indicated with an asterisk. (**C**) Heatmaps of the KEGG pathway functional enrichment analysis of DEGs during infection. Colors are representative of the enrichment significance [−log_10_(FDR)] scaled with the Z-score and indicated by up- (red) or downregulation (blue) of each pathway. The dendrogram illustrates the hierarchical clustering of pathways, while the samples were unclustered.

Several genes were upregulated at all timepoints, when compared to the *in vitro* culture control, but they were mostly stress-associated genes. There were some cell wall–associated proteins like *cwp10* (CDR20291_2685) [2.25 log_2_(FC) at 3 hours, *P* adj = 1.09E-09], CDR20291_0184 (putative cell wall hydrolase) [2.05 log_2_(FC) at 3 hours, *P* adj = 3.17E-08], and CDR20291_2686 [2.75 log_2_(FC) at 3 hours, *P* adj = 1.39E-012], which were highly upregulated at all timepoints after infection (Fig. 5A). Interestingly, the *cwp84* (CDR20291_2676), a cell wall hydrolase required for the formation of the S-layer was significantly downregulated [−2.25 log_2_(FC) at 3 hours, *P* adj = 4.30E-09] at all timepoints. *slpA* (CDR20291_2682), the precursor for the S-layer proteins, was also significantly downregulated at 6 hours post-infection (Fig. 5A). Several other CWPs, such as *cwp66* (CDR20291_2678), a CWP suggested to play a role in adhesion to Vero cells (14), *cwp13* (CDR20291_1645), *cwp14* (CDR20291_2624), *cwp17* (CDR20291_0892), *cwp18* (CDR20291_0903), *cwp19* (CDR20291_2655), and *cwp20* (CDR20291_1318) were downregulated at all timepoints after infection. *codY* (CDR20291_1115), a global transcriptional regulator (40), was significantly downregulated at 6, 12, and 24 hours after infection, and *fliC* (CDR20291_0240) at 12 hours post-infection. Proline-proline endopeptidase 1 (PPEP-1/CDR20291_2721), an important secreted zinc metalloprotease that cleaves cell-surface-associated collagen-binding proteins and host extracellular matrix proteins, was also significantly downregulated at 3 and 12 hours post-infection [1.12–1.79 log_2_(FC), *P* adj < 0.03] and was investigated further in this study (11, 41) (Fig. 5B).

Bacterial genes changing over time between 3 hours and other timepoints after infection were compared, and there were many metabolic pathway genes and few CWPs changing over time (Table 2). Like the host genes, we see some bacterial genes that are downregulated compared to the uninfected control but upregulated when compared across time (e.g., CDR20291_0892).

**TABLE 2:** Top 10 most significantly different bacterial genes changing at different times compared to 3 hours after infection

Time after infection (hours)	Gene	log_2_(FC)	*P* adj
3 vs 6	CDR20291_1591	3.58965355	4.00E-14
	*leuS*	−3.6691402	7.72E-11
	CDR20291_2824	2.05554368	9.22E-10
	CDR20291_0756	2.51963688	2.18E-09
	*rbo*	2.24137439	4.87E-09
	*rbr*	2.15269859	5.59E-09
	*glyA*	−2.1494402	5.59E-09
	*etfA1*	3.56674282	7.00E-09
	CDR20291_1118	2.67543257	7.00E-09
	CDR20291_2022	2.68921553	1.35E-08
3 vs 12	CDR20291_1591	4.25801398	9.87E-24
	CDR20291_2845	3.6460206	3.11E-20
	CDR20291_2824	2.15865642	2.49E-12
	*rbo*	2.38713598	9.17E-12
	*rbr*	2.26749276	2.05E-11
	CDR20291_1118	2.80879361	6.36E-11
	CDR20291_0756	2.49678131	1.27E-10
	CDR20291_0760	−3.1958283	1.65E-09
	CDR20291_2487	3.19721177	1.26E-08
	CDR20291_0318	2.59373112	1.73E-08
3 vs 24	CDR20291_1591	4.4613226	1.81E-26
	CDR20291_2487	4.97667554	7.12E-22
	CDR20291_1118	3.79570722	3.61E-21
	*rbo*	3.04052554	3.91E-20
	*etfA1*	4.77756376	4.49E-19
	CDR20291_0756	3.24899191	5.34E-19
	*hadA*	3.35378085	8.00E-19
	*hadC*	4.3369607	2.01E-18
	CDR20291_2845	3.39039969	6.75E-18
	*etfB1*	4.28467697	9.39E-18

To investigate bacterial pathways modulated during *C. difficile* infection, we performed a functional enrichment analysis of Kyoto Encyclopaedia of Genes and Genomes (KEGG) pathways. The up- or downregulated genes at each timepoint compared to the control were entered into the STRING database online tool to identify functional pathways enriched during *C. difficile* infection. There were many interesting significantly modulated pathways identified in this analysis, and most of these pathways were associated with the biosynthesis or metabolism of various substrates (Fig. 5C). The metabolic pathways of purines, pyrimidines, carbon, and methane were significantly upregulated during *C. difficile* infection. Interestingly, the biosynthesis of antibiotics, secondary metabolites, and gluconeogenesis pathways were also upregulated during host infection (Fig. 5C). The metabolic pathways of nitrogen, 2-oxocarboxylic acid, cysteine, methionine, butanoate, glycine, serine, threonine, propanoate, phenylalanine, fatty acid, pyruvate, carbon, glyoxylate, and dicarboxylate and the biosynthesis pathways of amino acids, secondary metabolites, aminoacyl-tRNA, and antibiotics were significantly downregulated during *C. difficile* infection (Fig. 5C). Some pathways, such as carbon metabolism and biosynthesis of secondary metabolites, were identified as both up- and downregulated, suggesting that these pathways were modulated, where some genes were upregulated and others were downregulated.

### Pyrimidine ribonucleotides were modulated during infection

**Fig 6:**
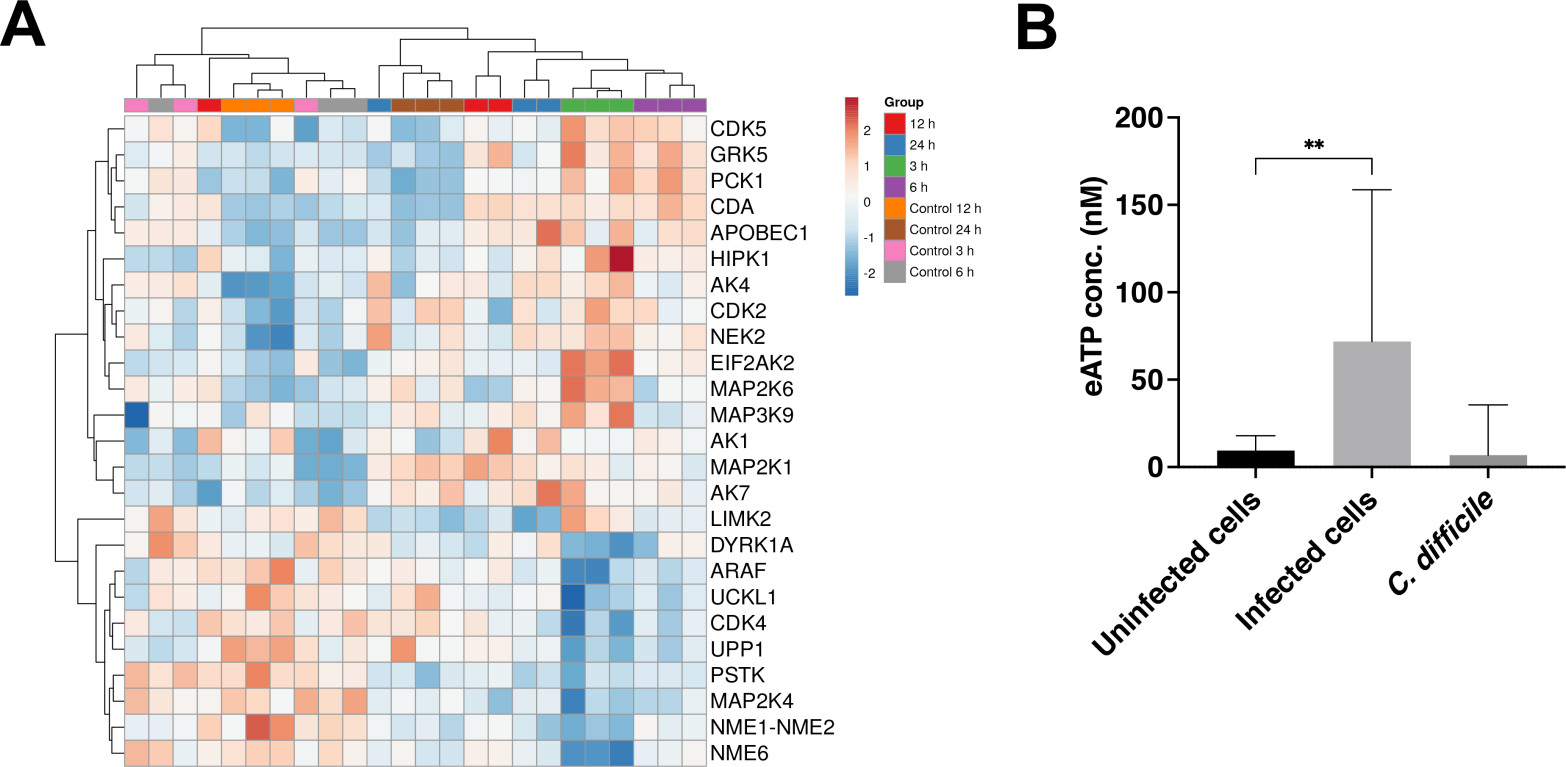
The differential expression of genes involved in the salvage pathways of pyrimidine ribonucleotides. (**A**) Heatmap of the genes involved in the salvage pathways of pyrimidine ribonucleotides at 3 hours post-infection. Regularized logarithmic transformation was applied to the normalized counts from DESeq2. The Z-score was calculated to scale the data, where red is upregulation and blue is downregulation. (**B**) ATP release assay from uninfected cells, *C. difficile*-infected cells, or a bacterial inoculum incubated in pre-reduced DMEM-10 for 4 hours under anaerobic conditions. Bars are representative of mean values from two independent experiments with nine technical replicates each, and error bars represent SD. **P = 0.007, by an unpaired *t*-test with Welch’s correction.

The most significantly modulated host pathway at 3 hours post infection was the salvage pathways of pyrimidine ribonucleotides, which was upregulated during infection. The normalized gene expression values of genes involved in the salvage pathways of pyrimidine ribonucleotides were plotted with a heatmap, and hierarchical clustering was applied to group genes and samples with similar gene expression profiles (Fig. 6A). In general, most of the samples from the four infected groups were clustered together more closely than the uninfected groups, although the 24-hour uninfected controls were clustered more closely to the infected sample groups than the other uninfected controls. At 3 hours post-infection, there was an upregulation of 12 genes and downregulation of 13 genes, which are shown in Fig. S5 in the context of this pathway. At the other three timepoints, there was also modulation of the genes involved in the salvage pathways for pyrimidine ribonucleotides, including cytidine deaminase (CDA), which was significantly upregulated at all timepoints (Fig. 6A).

In addition to the salvage pathways of pyrimidine ribonucleotides, there were several other significantly enriched pathways at 3 hours after infection with a role in the biosynthesis or metabolism of nucleotides including the nucleotide excision repair pathway, pyrimidine deoxyribonucleotides *de novo* biosynthesis I, purine nucleotides degradation II (aerobic), and adenosine nucleotides degradation II. Also, as noted from the bacterial gene analysis, purine and pyrimidine metabolic pathways were also significantly differentially expressed in *C. difficile*. To investigate whether extracellular nucleotides are involved in infection, we measured extracellular ATP (eATP), an interkingdom purinergic signaling molecule, which is an energy source for cells. A luminescence-based ATP release assay, where ATP is required for the conversion of luciferin into oxyluciferin, was used to quantify the amount of eATP released during *C. difficile* infection *in vitro*; the quantity of luminescence was directly proportional to the concentration of ATP in the reaction. ATP concentrations in supernatants from uninfected and *C. difficile*-infected intestinal epithelial cells were compared. At 4 hours post-infection, there was significantly more ATP released [71.83 nM (mean)] into the supernatant from *C. difficile*-infected intestinal epithelial cells compared to the uninfected cells [9.41 nM (mean)] (Fig. 6B). Interestingly, there was also a substantial amount of ATP released from the bacterial inoculum (Fig. 6B). This suggests that *C. difficile* may produce some of the eATP in the supernatant from infected cells.

### *∆PPEP-1* may play a role in *C. difficile* colonization of an intestinal epithelial layer

PPEP-1 is a secreted proline-proline endopeptidase that cleaves collagen-binding adhesins on the *C. difficile* cell surface, and mammalian proteins like fibrinogen (11, 41), although its role during infection is not clear. We hypothesized that PPEP-1 may be involved in the transition between sessile and motile lifestyles by releasing bacteria from the epithelial layer or ECM during infection through cleavage of the collagen-binding proteins CD2831 and CD3246. As the gene which encodes PPEP-1 was significantly downregulated at 3, 6, and 24 hours after infection, this may result in reduced cleavage of cell-surface-associated adhesins and consequently impact bacterial adhesion to host cells.

**Fig 7:**
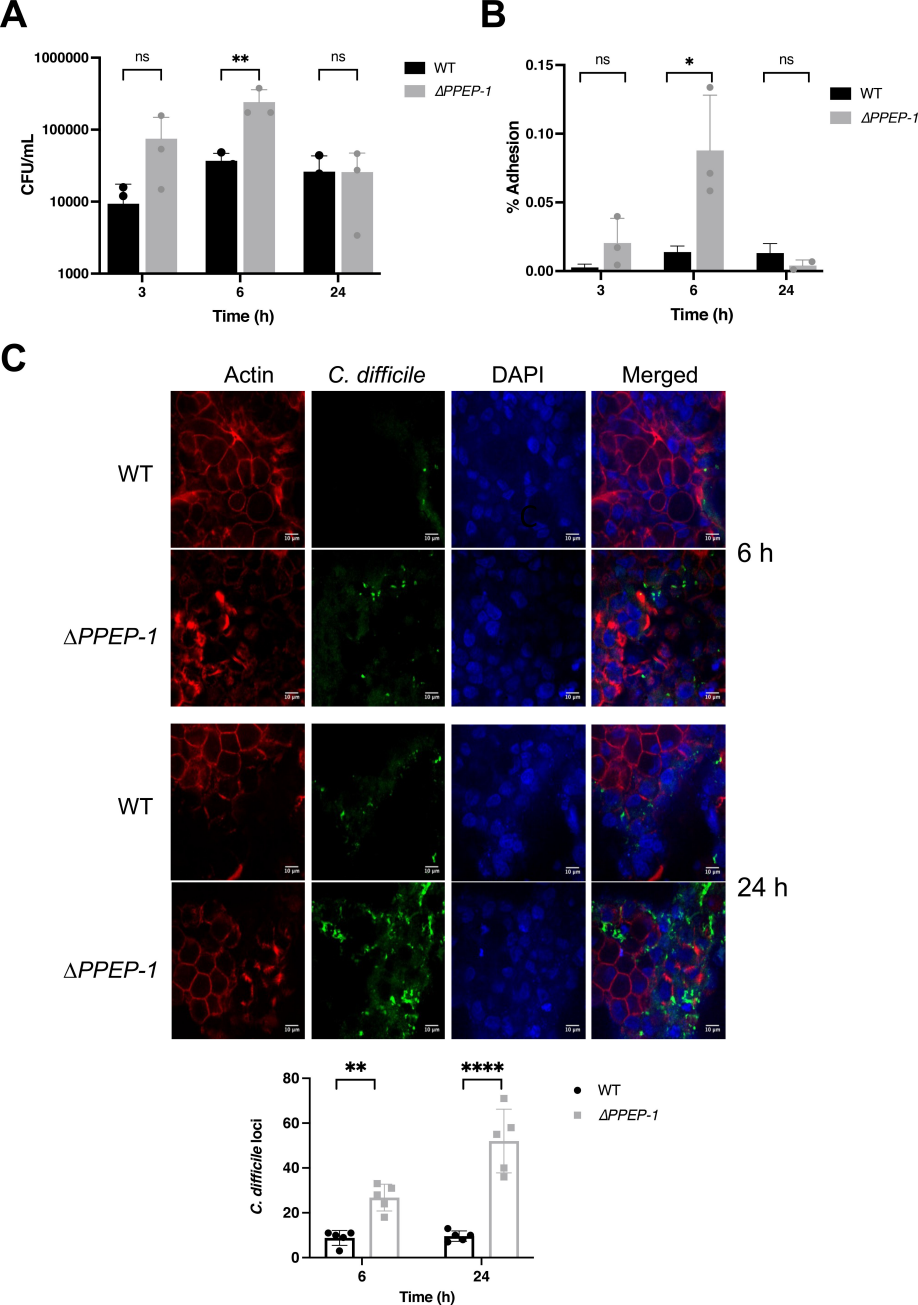
*∆PPEP-1* modulates bacterial adherence to intestinal epithelial cells. (**A**) The number of bacteria (CFU) attached to intestinal epithelial cells in the VDC system (*n* = 3) (**P = 0.003 at 6 hours), determined with two-way ANOVA (analysis of variance) with Sidak multiple comparisons test. (**B**) The percentage of adherent bacterial cells in relation to the inoculum was calculated using the equation (CFU of adhered cells/CFU of inoculum) × 100. Statistical significance was tested using an unpaired *t*-test, *P = 0.0342. Bars are representative of the mean ± SD. (**C**) Cell layers were stained with phalloidin (red), DAPI (blue), and anti-*C*. *difficile* antibody (green) and imaged with confocal microscopy. *C. difficile* cells adhered to the epithelial cell layers were counted. Statistical significance was tested by two-way ANOVA, **P = 0.0051, ****P < 0.0001. Data representative of three biological replicates, with five technical replicates (fields) analyzed. Bars are representative of the mean ± SD. ns, non-significant.

To investigate the role of PPEP-1 in *C. difficile* colonization, intestinal epithelial cells were infected with wild type (WT 630) or *∆PPEP-1* in the VDC system, and the number of bacteria attached to the epithelial cells was quantified by CFU counts. A higher number of *∆PPEP-1* bacterial cells adhered to the intestinal epithelial layers in VDCs compared to the WT 630 at 3 and 6 hours post-infection*,* while at 24-hour post-infection there was no significant difference in adhesion between the WT 630 and *∆PPEP-1* (Fig. 7A). Overall, while a low percentage of WT bacterial cells from the inoculum adhered to the epithelial cell layer at 6 hours post-infection, there was a significantly higher percentage of *∆PPEP-1* bacterial cells adhered to the epithelial cells compared to the WT (Fig. 7B). Additionally, in 24-well dish adhesion assays, we see a similar increase in adhesion for *∆PPEP-1*, with the percent adhesion reduced in the complemented strain (Fig. S6). Intestinal epithelial layers infected with WT 630 or *∆PPEP-1* in VDCs were also analyzed with confocal microscopy to compare the levels of bacterial adhesion. Quantitative analysis of the microscopy images confirmed that there were more *∆PPEP-1* bacterial cells attached to the epithelial cell layers compared to the WT at 6 and 24 hours post-infection (Fig. 7C). Thus, our data indicate that PPEP-1 is downregulated during the initial infection phase, which likely enables the activation of surface proteins that mediate host cell adhesion.

## DISCUSSION

*C. difficile* adhesion to the gut mucosa is crucial for the establishment of disease, yet we lack a comprehensive understanding of the host and bacterial factors required for colonization. Host-pathogen interactions of *C. difficile* infection, particularly in the initial phases of infection, have been largely understudied, mainly due to a paucity of controlled *in vitro* cellular models of infection. Here we report a transcriptomic analysis of epithelial cells infected by *C. difficile* in an *in vitro* gut model which supports co-culturing of anaerobic *C. difficile* with host cells. To our knowledge, this is the first dual RNA-seq study that has been conducted with an anaerobic microbe in the presence of human gut epithelial cells. Our data show that several host pathways were altered during the initial infection phase, including pyrimidine salvage pathways, modulation of cell wall–associated bacterial proteins, and a secreted protease, PPEP-1, which we demonstrate to have a role in mediating the adhesion of *C. difficile* to epithelial cells.

Dual RNA-seq, a relatively recent methodology, enables the simultaneous resolution of global transcriptomic profiles of both host and pathogen (42). New host and bacterial factors that modulate infection have been identified through dual RNA-seq for many aerobic bacterial pathogens, including uropathogenic *Escherichia coli* (UPEC) (43), *Mycobacterium tuberculosis* Bacillus Calmette–Guerin (BCG) (44), *Haemophilus influenzae* (45), and *Salmonella enterica* serovar Typhimurium (46). For example, dual RNA-seq of *Salmonella typhimurium*-infected HeLa cells identified the bacterial small RNA, PinT, as a regulator of genes required for intracellular survival and virulence. PinT was also reported to modulate the JAK/STAT signaling pathway in the host cells (46).

Previous transcriptomic studies have focused on the effects of *C. difficile* toxins on host cells. Inflammation-associated genes (such as C3, CCXL1, CXCL10, DUSP1, and EGR1) played a key role in the response to TcdA, TcdB, and a combination of both toxins (27). In a similar study investigating whole-tissue transcriptional responses to *C. difficile* R20291, the IL-1 cytokine family member, IL-33, was significantly upregulated during *C. difficile* infection (19). A recent study by Fletcher *et al.* examined the host transcriptomic responses to WT and *ΔtcdR C. difficile* in a mouse model, although only a targeted set of genes involved in immune responses were assessed using Nanostring analysis (28). Gene Ontology (GO) pathways that were differentially expressed during toxin-induced inflammation included the regulation of the inflammatory response, peptide secretion, and proteolysis. In terms of human transcriptomic responses to *C. difficile*, Caco-2 cells were infected with *C. difficile* for 30, 60, and 120 minutes in anaerobic conditions, and differentially expressed human genes were involved in a range of metabolic processes, such as nucleic acid, protein, and lipid metabolism, cell organization and biosynthesis, intracellular transport, cell communication, signal transduction, and transcription (38). In this study, we compared the transcriptomes of *C. difficile-*infected epithelial cells to uninfected cells at different timepoints over 24 hours post-infection, with cells oxygenated from the basolateral side, while exposed to anaerobic conditions on the apical side. It was evident from the changes seen in the uninfected control cells over time and the clustering of data, that although cells looked morphologically healthy and were in an intact layer, they were exposed to mild stress perhaps due to the apical exposure of anaerobic gas, particularly by 24 hours (31). However, distinct profiles were seen at different timepoints after infection, with maximal changes occurring at 3 hours, when the cells were first exposed to the bacteria (Fig. 3). While several metabolic pathways were seen to be modulated on infection, as reported previously (38), it was interesting to note an induction of the LRP and the Frizzled proteins, which are colonic receptors for *C. difficile* TcdA and TcdB (35, 47). During infection within this *in vitro* model, we see very low amounts of toxin A and toxin B produced during the initial 24 hours of infection, although this may be sufficient to trigger increased expression (31). Alternatively, the presence of *C. difficile* or other bacterial components may trigger the expression of these genes, which leads to the increased cellular binding and uptake of toxins.

Mucins have previously been implicated in *C. difficile* infection. Patients with CDI secrete mucus of altered composition in their feces, with higher levels of the membrane-bound mucin, MUC1, and decreased levels of secretory mucin MUC2, when compared to healthy patient stool (48). In this study, it was interesting that the membrane-bound immunomodulatory mucins 12 and 13 are upregulated during infection. MUC13 has been implicated in other microbial infections recently (49), and it would be interesting to explore the role of these immunomodulatory mucins during CDI further. In terms of immune pathways, IL-17 signaling pathways were significantly induced in epithelial cells early in infection, suggesting that this cytokine plays a key role in infection, as reported recently (25). There were some immune pathway–associated genes including genes from the TNF receptor superfamily (TNFRSF), which were downregulated during infection, which may suggest that the presence of the pathogen dampens TNFR-mediated cell signaling. Although transient upregulation in multiple matrix metalloproteinase (MMP) transcripts was reported previously during toxin-induced intestinal inflammation in mice (28), we see only MMP1 modulated over time in early infection. Overall, it is hard to directly compare the changes induced in the host transcriptome between studies due to differences in the types of host cells and models used, the timepoints post-infection, and the bacterial strains used.

Host pathways involved in the salvage of pyrimidine nucleotides were upregulated at all timepoints after infection. The modulation of this pathway during *C. difficile* infection is interesting because extracellular nucleotides have been reported to be regulators of the immune response (50). Bacteria have been reported to produce ATP during growth (51), and the eATP produced by intestinal bacteria in the gut can prevent the generation of a protective IgA response against invading bacterial pathogens (52). Our data suggest that *C. difficile* produces a low level of ATP. During infection, ATP produced by bacteria, as well as released by host cells may induce local inflammatory responses in the gut, and nucleotide salvage pathways may be triggered to recycle nucleotides, as well as contain the inflammatory responses.

There have been several studies examining the *C. difficile* transcriptome in different animal infection models. Using a microarray-based approach in a germfree mouse model, Janoir *et al.* reported that majority of the gene expression changes were in metabolism (fermentation, amino acid and lipid metabolism), stress response, pathogenicity, and sporulation, with the toxin genes *tcdA* and *tcdB* moderately upregulated at the later stages of infection (29). A study by Fletcher *et al*. analyzed the nutrients important during *C. difficile* VPI 10463 colonization using an RNA-seq approach. They showed that several metabolic pathways, including carbohydrate, amino acid, and fatty acid uptake and metabolism were upregulated early in the process of susceptible host colonization, with *feoB*, a ferrous iron importer being one of the top upregulated genes at the later stages of infection (53). In a microarray-based analysis of *C. difficile* responses in a pig ligated-loop model infection, upregulation of the *tcdA* was observed 12 hours post-infection, along with upregulation of colonization and virulence-associated genes, such as CD2830 (*PPEP-1*), CD2592 (fibronectin-binding protein), CD2793 (*slpA*), CD1546, CD1208 (hemolysins), and genes involved in the sporulation cascade (30). The present study highlights the modulation of several bacterial metabolic pathways, including fatty acid, amino acid, and nucleotide synthesis. The purine and pyrimidine synthesis pathways are initially upregulated, which may indicate an increase in nucleotide production, potentially enhancing an inflammatory response. Additionally, pyrimidine metabolism is known to be essential for *C. difficile* survival (54).

We observed several differentially expressed cell surface proteins, including SlpA, which were downregulated at all timepoints after infection relative to expression in the control medium (DMEM with 10% serum). When compared over time, that is, 3 hours to other timepoints, there are no changes in gene expression observed for *slpA*. The data suggest that the expression of these genes is perhaps dampened when the bacterium is in the presence of host cells. With the S-layer being an impervious tightly packed capsule around the bacterium (55), a potential explanation may be that its downregulation enables bacteria to secrete proteases or express surface proteins through S-layer-depleted regions of the cell wall. Another gene that was downregulated was *codY*, a global regulator of gene expression and suppressor of several virulence factors, such as toxin production and sporulation (40, 56). This regulator has been reported to be upregulated in another *in vitro* infection study, although the times and experimental conditions used in this study were different (38).

A downregulation of the proline-proline endopeptidase, PPEP-1, an enzyme that cleaves the bacterial surface adhesins CDR20291_2820 (CD630_28310) and CDR20291_2722 (CD630_32460), was also seen during infection (53). *∆PPEP-1* has been previously reported to exhibit an attenuation in virulence in a hamster model (41), although its precise role during infection was unclear. A previous study demonstrated that a PPEP-1 mutant showed enhanced binding to collagen-1, which CD630_28310 was also reported to bind to (57). The PPEP-1 protease is expressed when levels of intracellular c-di-GMP are low which activates the expression of motility-associated genes, such as flagella and chemotaxis genes (58), and the levels of PPEP-1 substrates, CD2831 and CD3246, are higher when c-di-GMP levels are high, and the bacteria are in an adhesive state (59). Here, we demonstrate a role for PPEP-1 as a negative modulator of epithelial cell attachment during *C. difficile* infection, suggesting that this enzyme may have a role in the release of *C. difficile* from intestinal epithelial cell layers, through cleavage of cell surface adhesins, aiding *C. difficile* dispersal and penetration of gastrointestinal tissues.

Thus, we describe transcriptional changes occurring in human epithelial cells and in the bacterium during the initial contact between *C. difficile* with host cells within an *in vitro* gut infection model. While the *in vitro* model described in this study mimics the gut environment to some extent, including small amounts of mucus, it lacks the thick complex mucus layer that overlays the gut epithelium. Additionally, the host transcriptional changes elicited may be common to those in response to other anaerobic bacteria; further comparisons with other gut pathogens and commensals are necessary to delineate *C. difficile*-specific responses. Nevertheless, this analysis sheds light on the genes and pathways which are modulated during the early host association of *C. difficile*, providing new insights into potential mechanisms of *C. difficile*-host interactions.

## MATERIALS AND METHODS

### Bacterial strains and growth conditions

A B1/NAP1/027R20291 (isolated from the Stoke Mandeville outbreak in 2004 and 2005) was used for the dual RNA-seq studies. *Clostridioides difficile* strain 630 was used for the studies on PPEP-1. *ΔPPEP-1* mutant (in a *C. difficile* 630 background) was provided a kind gift from Prof. Neil Fairweather, Imperial College London. Strains used in this study are included in Table S6. *C. difficile* strains were streaked from glycerol stocks and cultured using brain-heart infusion (BHI) agar or broth (Sigma Aldrich, USA) supplemented with 1 g/L l-cysteine (Sigma Aldrich, USA) and 5 g/L yeast extract (Sigma Aldrich, USA) (BHIS) under anaerobic conditions (80% N_2_, 10% CO_2_, 10% H_2_) in a Don Whitley workstation (Yorkshire, UK).

### Generation of the *∆PPEP-1* complemented strain

CD630_28300 (PPEP-1) was amplified using the forward primer (CTGAGCTCCTGCAGTAAAGGAGAAAATTTTATGAGACCAAGTAAAAAATT) and reverse primer (TAGGATCCGGCTATTTAGCTAAATTTTGCA), cloned into the BamHI and SacI restriction sites of a pRPF185 vector (Addgene, USA) and transformed into the conjugation donor strain *E. coli* CA434. Stationary overnight culture of the conjugative donor strain *E. coli* CA434 pRPF185-PPEP-1 was centrifuged, washed, re-suspended in 200 µL stationary overnight culture of *∆PPEP-1*, and this conjugation mixture was incubated for 24 hours on non-selective BHIS agar. Following incubation, all cells were harvested and re-streaked on *C. difficile* selective BHIS agar (supplemented with d-cycloserine 250 µg/mL, Cefoxitin 8 µg/mL, and thiamphenicol 15 µg/mL), incubated under anaerobic conditions at 37°C for 24–72 hours. Single colonies were isolated and re-streaked; the plasmid was extracted and confirmed by Sanger sequencing.

### Cell culture, media, and conditions

Intestinal epithelial cell line, Caco-2 (P6-P21) from American Type Culture Collection, mucus-producing cell line, HT29-MTX (P45-P60), a kind gift from Prof. Nathalie Juge, Quadram Institute, Norwich, and intestinal myofibroblast cells, CCD-18co (P10–P20) were used in this investigation. Caco-2 cells were grown in Dulbecco’s modified Eagle’s medium (DMEM) supplemented with 10% fetal bovine serum (FBS) (DMEM-10) (Labtech, United Kingdom), and 1% penicillin-streptomycin (10,000 units/mL penicillin, 10 mg/mL streptomycin; Sigma Aldrich, USA). HT29-MTX were grown in DMEM-10 and CCD-18co in Eagle’s Minimum Essential Medium media, both supplemented with 10% FBS, 1% penicillin-streptomycin, 2 mM glutamine, and 1% nonessential amino acids (Sigma Aldrich, USA). All cell lines were maintained in 5% CO_2_ in a humidified incubator at 37°C and free from mycoplasma contamination as determined on a regular basis by the EZ-PCR Mycoplasma kit (Biological Industries, USA). Snapwell inserts (Scientific Laboratory Supplies Ltd, United Kingdom) were prepared as described in the reference (31). Briefly, prior to seeding cells, the Snapwell inserts were coated with a 1:1 ratio of rat tail collagen (Sigma Aldrich, USA) and ethanol (VWR Chemicals, USA). Caco-2 and HT29-MTX were mixed in a 9:1 ratio and 2 × 10^5^ cells total was seeded on 12 mm Snapwell inserts (tissue culture treated polyester membrane, Corning, USA) for ~2 weeks to form a polarized monolayer. Caco-2 and HT29-MTX were seeded on the apical side of the Snapwell insert for 14 days. Prior to infection experiments, the cell culture medium in the Snapwell inserts was replaced with antibiotic-free medium 24 or 48 hours before the start of infection.

### Infection of intestinal epithelial cells in the VDCs

The Snapwell inserts containing the polarized cell layers were placed between the two half chambers of the VDC (Harvard Apparatus, Cambridge, United Kingdom) and sealed with the provided clamps. DMEM-10 of 3 mL was added to both sides to fill the chamber. As described previously (31), a single bacterial colony was inoculated in pre-reduced BHIS broth and incubated at 37°C for 16 hours in anaerobic conditions. The culture was centrifuged at 5,000 rpm for 5 minutes (Eppendorf 5810R; Eppendorf, Germany), and the bacterial pellet was resuspended in DMEM-10, before being diluted to an OD_600_ of 1.0 and incubated at 37°C in anaerobic conditions for 1 hour. Bacterial cultures were added in the anaerobic (apical) chamber such that intestinal epithelial cells were infected with an MOI of 100:1. This chamber was supplied with anaerobic gas mixture (10% CO_2_, 10% H_2_, 80% N_2_; BOC, United Kingdom) and the basolateral compartment with 5% CO_2_ and 95% air (BOC, United Kingdom) at a rate of ~1 bubble every 5 seconds. At 3 hours post-infection, the apical media containing *C. difficile* was removed, the chamber was washed once with phosphate-buffered saline (PBS), and 3 mL of fresh pre-reduced DMEM-10 was added. Chambers were incubated for a further 3–24 hours. The intestinal epithelial cell layers were washed thrice with pre-reduced PBS before being lysed with 1 mL of sterile water. The number of cell-associated bacteria was quantified by performing serial dilutions from the intestinal epithelial cell lysate and determining CFUs on BHIS agar.

### Infection/adhesion assay in 24-well dishes

Caco-2 and HT29-MTX were mixed in a 9:1 ratio and 2 × 10^5^ cells total were seeded in 24-well tissue culture– treated polystyrene plates (Costar, USA) and maintained for ~2 weeks to allow for the formation of a polarized monolayer. The day prior to the infection experiment, the medium was replaced with antibiotic-free medium. Overnight cultures of *C. difficile* were centrifuged at 10,000 × *g* for 5 mins, resuspended in DMEM-10, diluted to OD_600_ 1.0 in pre-reduced DMEM-10, and incubated at 37°C for 1 hour. One millliliter of this culture was incubated with cells in anaerobic conditions for 3 hours. Cells were washed once with PBS, followed by the addition of 1 mL of fresh DMEM-10, and incubated for a further 3 hours. Cells were washed twice with pre-reduced PBS and lysed with 1 mL of sterile water. The number of cell-associated bacteria were quantified by performing serial dilutions from the intestinal epithelial cell lysates and plating on BHIS agar to determine CFU/mL.

### RNA isolation

Bacterial and human cells were treated with RNAprotect cell or bacterial reagent (Qiagen, Germany) and stored at −80°C for up to 1 week. To extract RNA, samples were thawed and centrifuged at 4°C (Sigma Zentrifugen 1-14K, Germany). Cell pellets were resuspended in 1 mL of buffer RLT from the RNeasy mini kit (Qiagen, Germany) with a 1:200 dilution of β-mercaptoethanol (Sigma Aldrich, USA). Cells were homogenised using lysing matrix B tubes with 0.1-mm silica beads (MP Biomedicals, USA) in a FastPrep-24 5G (6.5 m/second for 20 seconds with 180 seconds rest on ice for six cycles) (MP Biomedicals, USA). RNA was extracted using the RNeasy RNA isolation kit (Qiagen, Germany), according to the manufacturer’s protocol. A rigorous treatment with TURBO DNase (Thermo Fisher Scientific, USA) was used to remove genomic DNA contamination from RNA samples, and clean-up was performed using the RNeasy mini kit following the manufacturer’s protocol (Qiagen, Germany). RNA concentrations were quantified using the Qubit RNA BR assay kit (Thermo Fisher Scientific, USA). The RNA quality was examined using the Bioanalyzer RNA 6000 pico kit (Agilent, USA).

### Ribosomal RNA depletion, library preparation, and RNA sequencing

An RNase H method for rRNA depletion was used as described by Adiconis *et al.* (2013). The human rRNA single-stranded DNA probes were purchased as oligonucleotides (Integrated DNA Technologies, USA). *C. difficile* probes complementary to the 16S, 23S, and 5S rRNA sequences were designed as ~50 bp non-overlapping primers (Integrated DNA Technologies, USA). RNase H rRNA depletion protocol was used as described by SciLifeLab (Sweden). Briefly, oligonucleotide probes were hybridized to RNA samples by incubation with hybridization buffer (1 M NaCl, 0.5 M Tris-HCl pH 7.4) in a Bio-Rad T100 thermocycler (95°C for 2 minutes, temperature decreased −0.1 C/s to 45°C and hold at 45°C). Thermostable RNase H (New England Biolabs, USA) was added, and the reaction was incubated at 45°C for 30 minutes. Agencourt AMPure XP magnetic beads were used to purify RNA. DNase I (New England Biolabs, USA) was used to remove leftover DNA oligonucleotides, and Agencourt AMPure XP magnetic beads (Beckman Coulter Life Sciences, USA) were used to clean up rRNA-depleted samples. Bioanalyzer RNA pico analysis was used to assess the RNA quality and effectiveness of the rRNA depletion (Agilent, USA). The NEBNext Ultra II Directional RNA Library Prep Kit for Illumina with the NEBNext Multiplex Oligos for Illumina (Index Primers Set 1) were used to generate DNA libraries for sequencing (New England Biolabs, USA) as per the manufacturer’s protocol. Agencourt AMPure XP magnetic beads (Beckman Coulter Life Sciences, USA) were used for bead-based DNA clean-up. Quality of DNA libraries was assessed using a Bioanalyzer High Sensitivity DNA kit (Agilent, USA). Single-end sequencing was performed using a NextSeqR 500/550 High Output Kit v2 (75 cycles) on an Illumina NextSeq500 system (Illumina, USA). Bacterial controls were sequenced using single-end sequencing with a MiSeq v3 cartridge (150 cycles) on an Illumina MiSeq system.

### RNA-seq analysis

Raw sequencing files were converted to FASTQ files using the bcl2fastq Linux software package (version 2.20), and the quality of the FASTQ files was assessed using the FastQC Linux package (version 0.11.9). FASTQ files were mapped to the appropriate reference genome (FN545816.1 for *C. difficile* R20291 and GRCh38 for humans) using HISAT2 (version 2.1.0). SAM files were converted to sorted BAM files and sorted using the Samtools (version 1.3.1) ‘view’ and ‘sort’ functions. The abundance of each genomic feature was analyzed using HTseq-count (version 0.11.2). The data were analysed in R (version 3.6.0) and were filtered to only include genes that had >10 reads in >10 samples. The DESeq2 R package (version 1.36.0) was used to calculate the differential gene expression profile using a negative binomial distribution model. Differences between groups were considered significantly different where the adjusted *P*-value was below 0.05 and log_2_(FC) was greater than 1 or less than −1 for human and bacterial samples. The ‘ggplot2’ R package (version 3.3.6) was used to generate data visualizations, including principal component analysis (PCA) plots, Venn diagrams, etc. Heatmaps were generated using the ‘pheatmap’ R package (version 1.0.12), where regularized logarithmic transformation was applied to the normalized counts from DESeq2, and gene expression data were scaled using the Z-score. For hierarchical clustering, row and column clustering distance were calculated with the Euclidean distance and clustered with the average linkage method. Volcano plots were generated with the ‘EnhancedVolcano’ R package (version 1.14.0).

### Pathway and functional analyses

Host pathway and functional analyses were performed using Ingenuity Pathway Analysis (IPA) software (QIAGEN, USA). The lists of differentially expressed genes from the host and bacteria were uploaded to IPA software and analyzed for enrichment of gene annotation terms to obtain a list of canonical pathways potentially modulated during host-bacteria interaction. For these analyses, Fisher’s exact test was used to measure the enrichment significance based on the number of molecules/genes overlapping each identified pathway. For the bacterial analysis, sequencing reads were aligned to the *C. difficile* strain 630 genome, analyzed for differential expression, and DEGs were analyzed for enrichment using the STRING online database (https://string-db.org) to investigate bacterial KEGG pathways and protein-protein interaction networks.

### ATP release assay

The OD_600_ of *C. difficile* R20291 overnight cultures grown in BHIS were resuspended in pre-reduced DMEM-10, diluted to an OD_600_ of 1 and cultures were incubated for 1 h. 1 mL of each culture was added to intestinal epithelial cells cultured as described above in a 24-well plate. Inoculum tubes kept as uninfected bacterial controls and 1 mL of pre-reduced DMEM-10 was added to half of the intestinal epithelial cells to be uninfected cell controls. After the required amount of time, supernatants from infected cells or bacterial cultures were filter sterilised through a 0.2 micron syringe filter and extracellular ATP (eATP) was quantified using the CellTiter-Glo® Luminescent Cell Viability Assay (Promega, USA) according to the manufacturer’s protocol. This assay can detect ATP from <10 cells (~1 μM). Luminescence was measured by a FLUOstar Omega Multimode Microplate Reader (BMG Labtech, Germany).

### Immunofluorescent staining and confocal microscopy analysis

Epithelial cell layers infected with *C. difficile* as described above were washed thrice with PBS to remove unadhered bacteria and fixed with 4% paraformaldehyde (PFA) (Alfa Aesar, USA) for 15 minutes at RT. Cells were permeabilized with 1% saponin (Sigma Aldrich, USA) in 0.3% Triton X- 100 (Sigma Aldrich, USA) in PBS (Thermo Fisher Scientific, USA) and then blocked with 3% bovine serum albumin (BSA) (Sigma Aldrich, USA) in PBS. Rabbit anti-*C*. *difficile* sera was used as a primary antibody to stain *C. difficile* on infected intestinal epithelial cells (1:500 dilution in 1% BSA in PBS for 1 hour at RT), followed by goat anti-rabbit IgG Alexa Fluor 488 conjugate antibody (1:200 dilution in 1% BSA in PBS for 1 hour at RT in the dark) (Cell Signalling Technology, USA) as the secondary antibody. Alexa Fluor 647 phalloidin (Cell Signalling Technology, USA) was used at a 1:100 dilution in PBS to stain the actin cytoskeleton. ProLong Gold Antifade Reagent with DAPI (Cell Signalling Technology, USA) was used to stain cell nuclei and seal coverslips. Slides were imaged using a confocal spinning-disk microscope (VOX UltraView; PerkinElmer, USA) with a 40× oil objective and two Hamamatsu ORCA-R2 cameras by Volocity 6.0 (PerkinElmer, USA). Image analysis was performed using the Fiji software package (version 2.0.0).

## Data Availability

All sequencing reads were deposited to the European Bioinformatics Institute ArrayExpress (accession number E-MTAB-12660).

## SUPPLEMENTAL MATERIAL

Supplemental figures msystems.00555-23-s0001.pdf Figures S1-S6.

Supplemental tables msystems.00555-23-s0002.pdf Tables S1, S2, and S6 and captions for other tables.

Table S3 msystems.00555-23-s0003.xlsx All significant DEGs from all time points compared to control for bacterial and human samples.

Table S4 msystems.00555-23-s0004.xlsx All significant DEGs from all time points compared to 3 h for bacterial samples.

Table S5 msystems.00555-23-s0005.xlsx All significant DEGs from all time points compared to 3 h for human samples.

ASM does not own the copyrights to Supplemental Material that may be linked to, or accessed through, an article. The authors have granted ASM a non-exclusive, world-wide license to publish the Supplemental Material files. Please contact the corresponding author directly for reuse.
