# Supplementary material for: Dual RNA-seq identifies genes and pathways modulated during Clostridioides difficile colonization
Source: mSystems. 2023 Aug 24;8(5):e00555-23. doi: 10.1128/msystems.00555-23 (PMC10654110; doi:10.1128/msystems.00555-23)
Supplement: Supplemental figures — Figures S1-S6. [file msystems.00555-23-s0001.pdf]

Figure S1

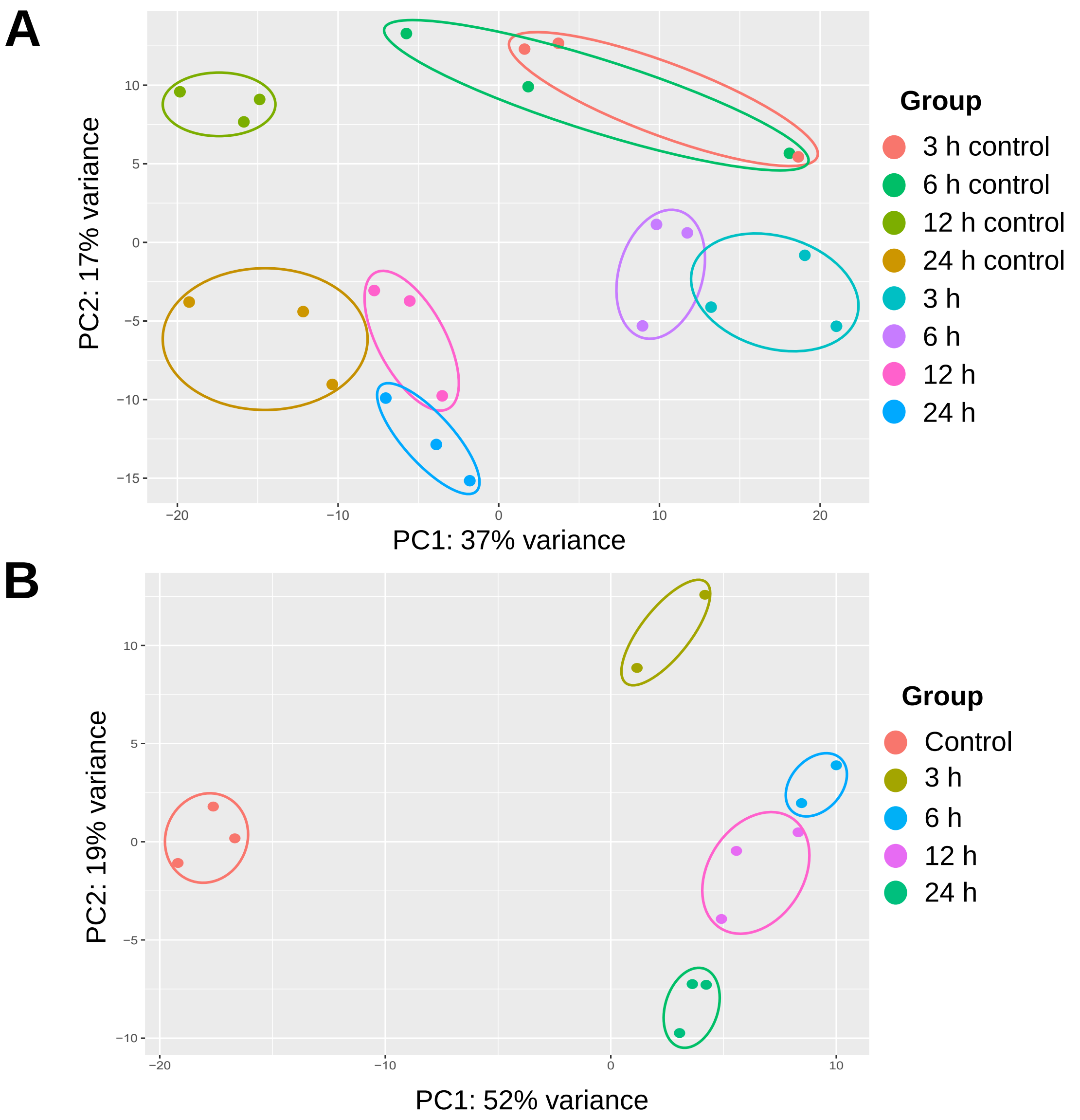

Figure S1. A. PCA plot of host RNA-seq data to show sample-sample distances of infected samples and uninfected controls at 3, 6, 12 and 24 h. B. PCA plot of bacterial RNA-seq data to show sample-sample distances of infected samples at 3, 6, 12 and 24 h and an uninfected control culture.

Figure S2

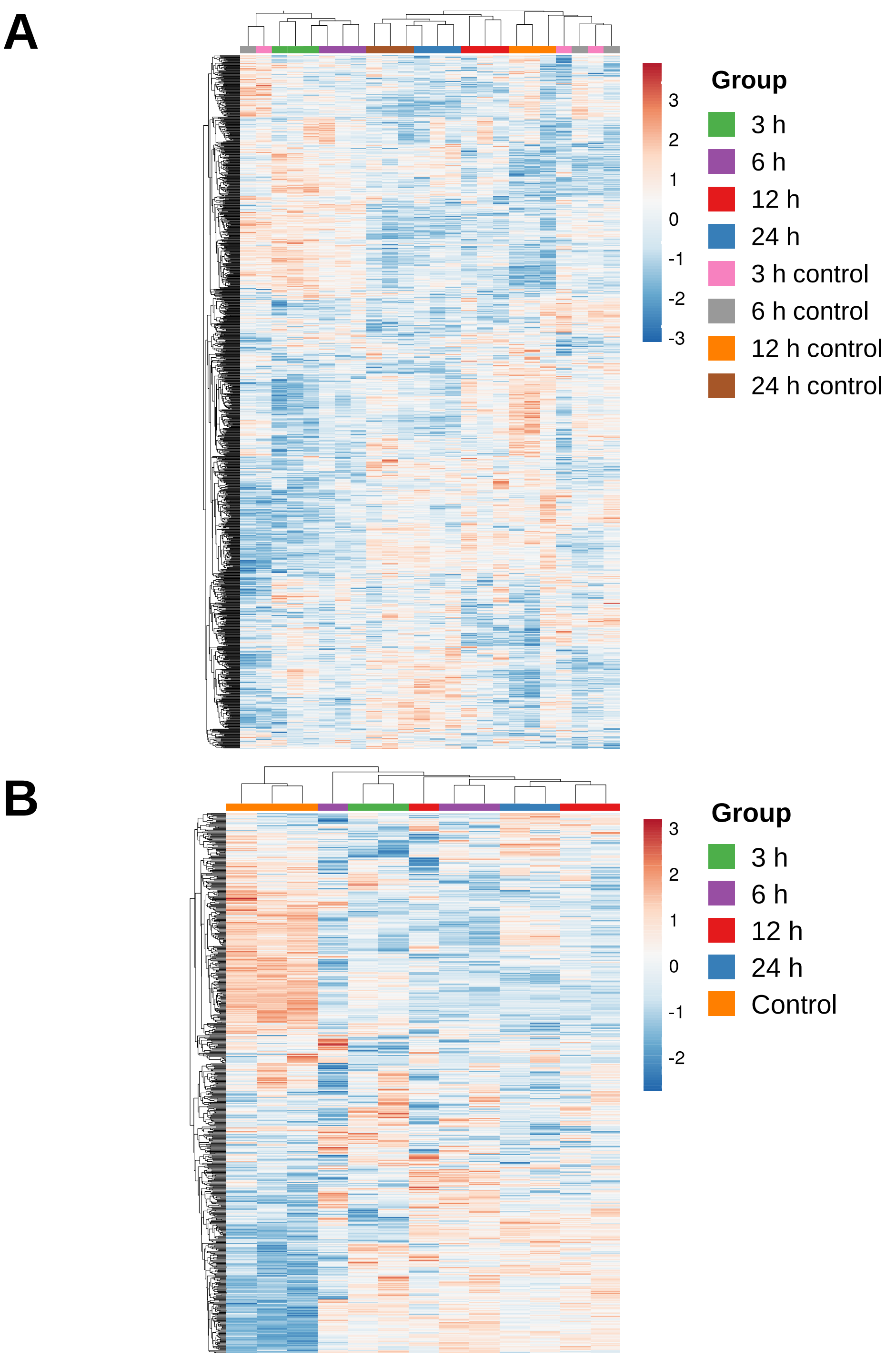

Figure S2. Heatmaps of host and bacterial transcriptomic data. A. Heatmap of host transcriptomic data to demonstrate the variations in gene expression between the infected samples and uninfected controls at each timepoint. B. Heatmap of bacterial transcriptomic data to demonstrate the variations in gene expression between the infected samples and uninfected controls at each timepoint. Gene expression values were scaled using the Z-score, which is represented with a colour scale, where red indicates a positive Z-score, and blue indicates a negative Z-score.

Figure S3

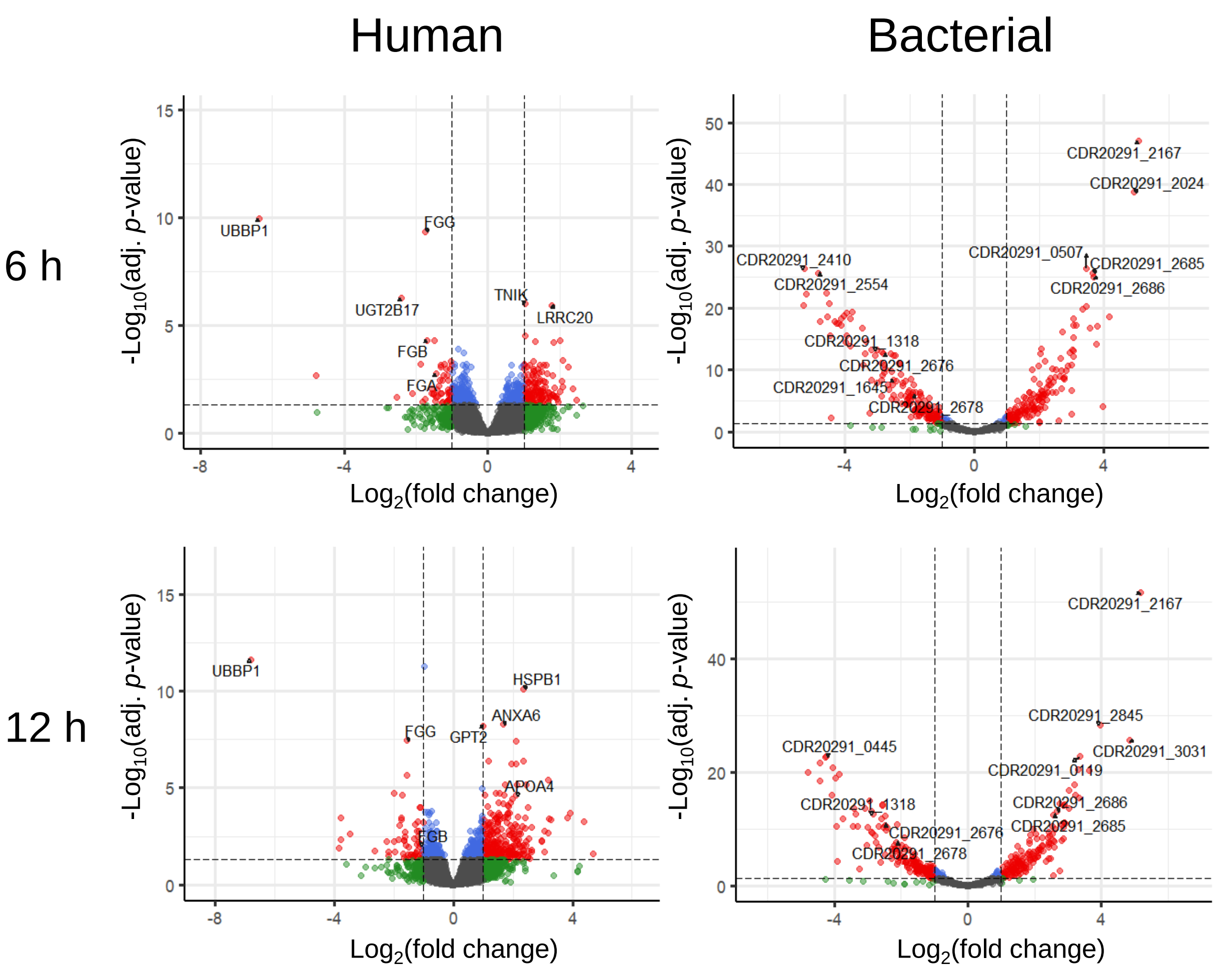

Figure S3. Volcano plots to illustrate the distribution of human and bacterial differentially expressed genes between uninfected controls and infected samples at 6 h and 12 h after infection. Significantly differentially expressed genes can be visualised as having a $\log_2$(FC) greater than 1 or less than -1 (vertical lines) and adjusted *p*-value less than 0.05 (horizontal line). Arrows point out selected highly significantly differentially expressed genes.

Figure S4

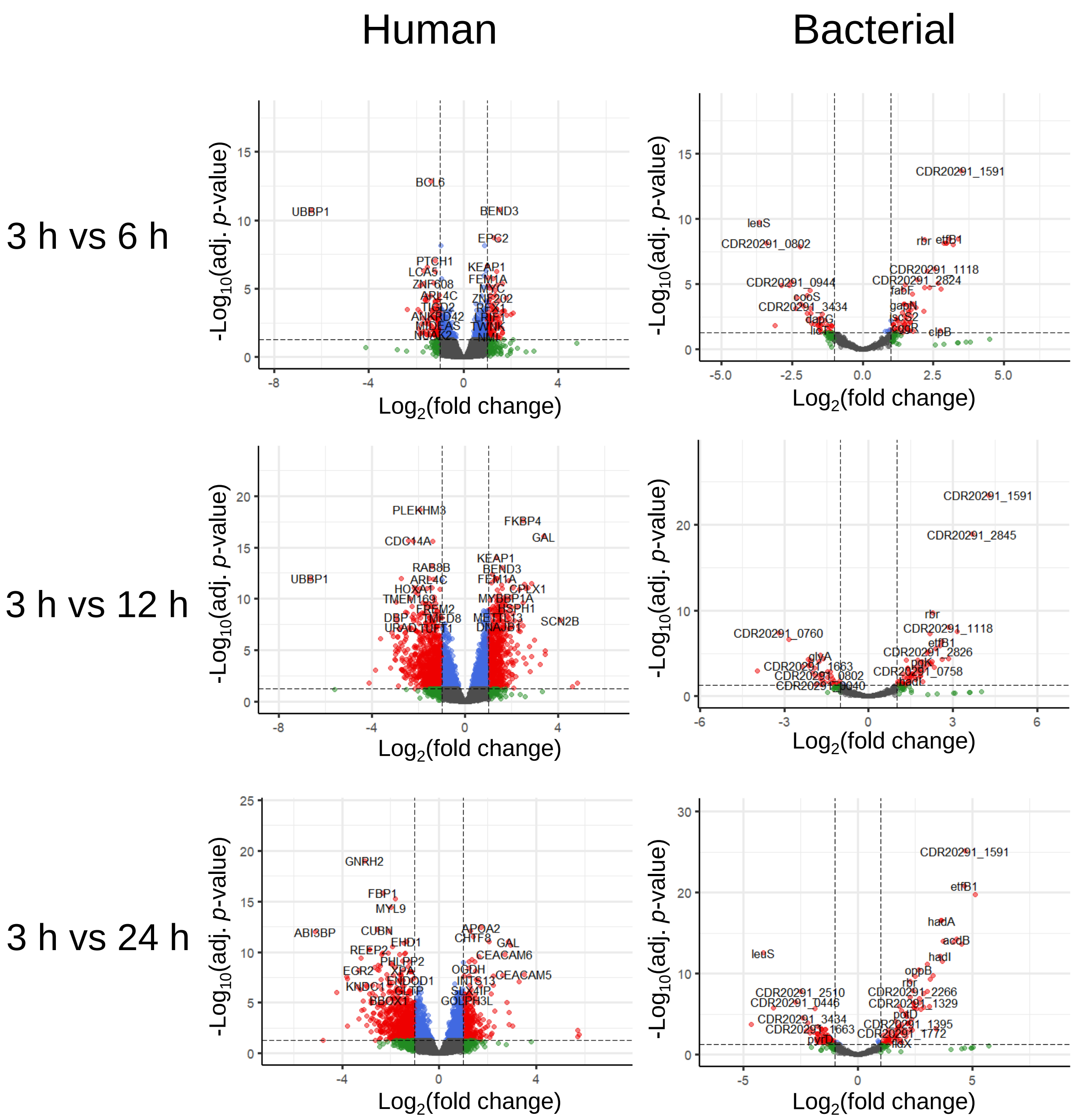

Figure S4. Volcano plots to illustrate the distribution of human and bacterial differentially expressed genes of infected samples between 3 h and 6, 12 or 24h after infection. Significantly differentially expressed genes can be visualized as having a $\log_2(FC)$ greater than 1 or less than -1 (vertical lines) and adjusted p-value less than 0.05 (horizontal line). Arrows point out selected highly significant DEGs.

Figure S5

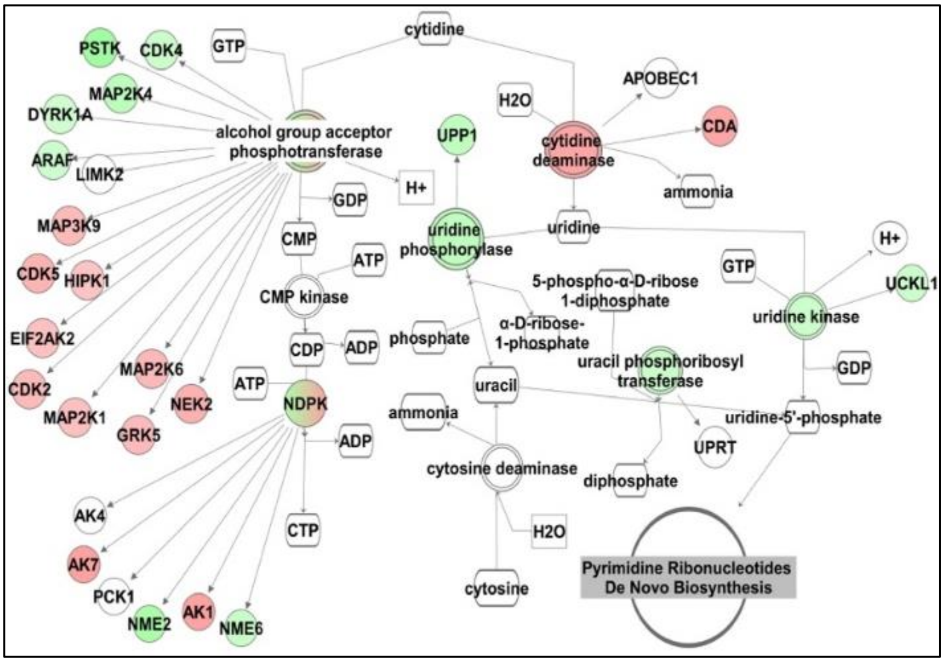

Figure S5. A pathway map of the pyrimidine ribonucleotide *de novo* biosynthesis KEGG pathway. Upregulated genes are coloured in red and downregulated genes are coloured in green.

Figure S6

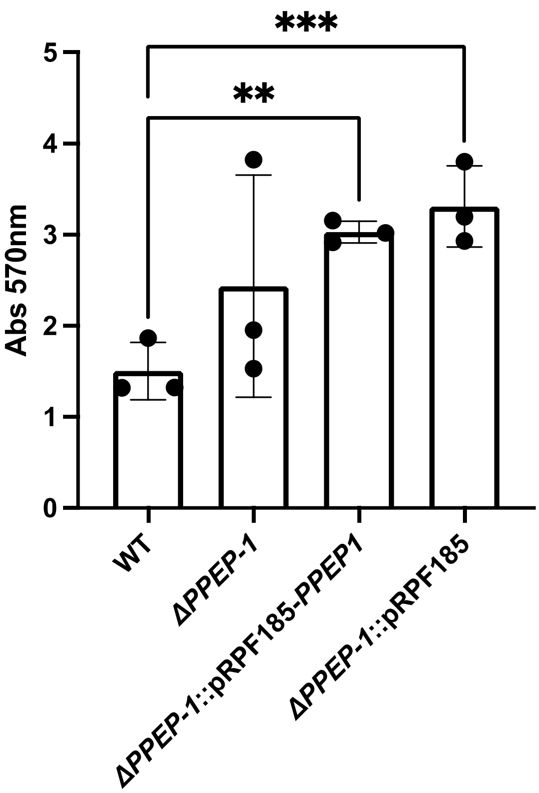

Figure S6. PPEP-1 does not impact biofilm formation. Biofilm biomass formed by *C. difficile* strains WT 630, Δ*PPEP-1*, and complemented strain Δ*PPEP-1+ pRPF185-PPEP-1 or* plasmid control Δ*PPEP-1* + *pRPF185* over 24 h in BHIS+G as quantitated by crystal violet staining. Bars are representative of the mean +/- standard deviation. ****p*< 0.001, ***p*<0.01 indicates significant differences, calculated using a one-way ANOVA multiple comparison test.

Figure S7

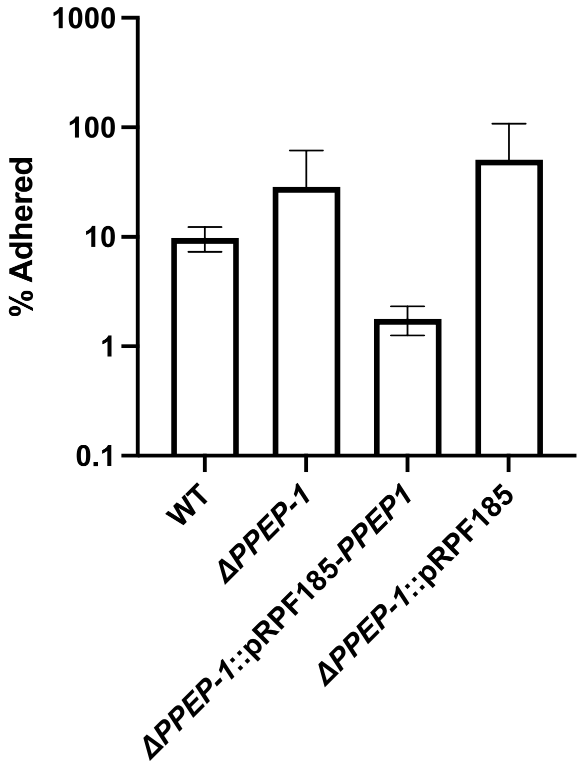

Figure S7. The impact of PPEP-1 on adhesion. Intestinal epithelial cells were incubated for 3 h with *C. difficile* strains WT 630, Δ*PPEP-1*, and complemented strain Δ*PPEP-1* + pRPF185-*PPEP-1* or plasmid control Δ*PPEP-1* + pRPF185 at an MOI of 100:1 in a 24-wells, washed and incubated further for 3 h. Percentage of bacteria adhered from the initial inoculum was calculated using the equation (CFU of adhered bacteria/CFU of inoculum) x 100. Data representative of 2 biological replicates, with 3 technical replicates. Bars are representative of the mean +/- standard deviation.
