# Supplementary material for: Dual RNA-seq identifies genes and pathways modulated during Clostridioides difficile colonization
Source: mSystems. 2023 Aug 24;8(5):e00555-23. doi: 10.1128/msystems.00555-23 (PMC10654110; doi:10.1128/msystems.00555-23)
Supplement: Supplemental tables — Tables S1, S2, and S6 and captions for other tables. [file msystems.00555-23-s0002.pdf]

Table S1. Transepithelial electric resistance (TEER) measured in the uninfected control cells incubated in VDC's for different times (run in parallel to the cells harvested for RNA extraction)

| | | | | |
|---|---|---|---|---|
| TEER (mean ohm ± standard deviation) | 656.33 ± 109.60 | 617.33 ± 64.57 | 513.33 ± 147.31 | 420 ± 66.14 |

**Table S2 Total number of reads, percentage of reads aligned to a concatenated dual reference genome or *C. difficile* genome of all sequenced samples.**

| Sample type | Number of reads | Aligned to concatenated genome reference (%) | Aligned to *C. difficile* genome (%) |
|---|---|---|---|
| **Bacterial control 1** | 8936289 | 99.11 | 99.21 |
| **Bacterial control 2** | 12094137 | 99.02 | 99.40 |
| **Bacterial control 3** | 70755533 | 99.33 | 99.55 |
| **Human control 3 h 1** | 19115353 | 94.38 | 0.00 |
| **Human control 3 h 2** | 25470128 | 90.68 | 0.00 |
| **Human control 3 h 3** | 27025069 | 95.67 | 0.00 |
| **Human control 6 h 1** | 21468597 | 91.70 | 0.00 |
| **Human control 6 h 2** | 24593073 | 92.11 | 0.00 |
| **Human control 6 h 3** | 57975499 | 96.20 | 0.00 |
| **Human control 12 h 1** | 22390842 | 91.97 | 0.00 |
| **Human control 12 h 2** | 23882910 | 94.54 | 0.00 |
| **Human control 12 h 3** | 58915160 | 96.13 | 0.00 |
| **Human control 24 h 1** | 23835563 | 90.63 | 0.00 |
| **Human control 24 h 2** | 21564602 | 93.29 | 0.00 |
| **Human control 24 h 3** | 45215959 | 94.42 | 0.00 |
| **3 h infected 1** | 49226860 | 95.45 | 1.32 |
| **3 h infected 2** | 58246564 | 97.60 | 0.19 |
| **3 h infected 3** | 55316199 | 96.99 | 1.68 |
| **6 h infected 1** | 57700752 | 97.21 | 0.14 |
| **6 h infected 2** | 58987014 | 98.14 | 0.06 |
| **6 h infected 3** | 57515435 | 97.75 | 0.32 |
| **12 h infected 1** | 56507425 | 97.25 | 0.33 |
| **12 h infected 2** | 53660909 | 98.25 | 0.11 |

| | | | |
|---|---|---|---|
| **12 h infected 3** | 53241414 | 97.93 | 0.42 |
| **24 h infected 1** | 62622140 | 97.76 | 2.06 |
| **24 h infected 2** | 64673740 | 97.50 | 0.06 |
| **24 h infected 3** | 54650378 | 96.98 | 0.44 |

**Table S3.** Excel file with all significant DEGs from all timepoints compared to control for bacterial and human samples.

**Table S4.** Excel file with all significant DEGs from all timepoints compared to 3 h for bacterial samples.

**Table S5.** Excel file with all significant DEGs from all timepoints compared to 3 h for human samples (DEGs changing in uninfected control samples were removed).

Table S6. *C. difficile* strains used in this study.

| Strain | Description | Source |
|---|---|---|
| R20291 | Wild type strain B1/NAP1/027 R20291 | Isolated from the Stoke Mandeville outbreak in 2004 and 2005 |
| 630 | Wild type strain 630 | (1) |
| *630ΔPPEP-1* | *PPEP-1* deletion mutant in strain 630 | (1) |
| *630ΔPPEP-1::pRPF185* | *PPEP-1* deletion mutant with expression plasmid | This study |
| *630ΔPPEP-1::pRPF185-PPEP-1* | *PPEP-1* complemented strain | This study |

References

1. Peltier J, Shaw HA, Couchman EC, Dawson LF, Yu L, Choudhary JS, Kaever V, Wren BW, Fairweather NF. 2015. Cyclic diGMP regulates production of sortase substrates of *Clostridium difficile* and their surface exposure through ZmpI protease-mediated cleavage. J Biol Chem 290:24453-69.
